# Moldrug algorithm for an automated ligand binding site exploration by 3D aware molecular enumerations

**DOI:** 10.1186/s13321-025-01022-3

**Published:** 2025-05-26

**Authors:** Alejandro Martínez León, Benjamin Ries, Jochen S. Hub, Aniket Magarkar

**Affiliations:** 1 Theoretical Physics and Center for Biophysics, Universität des Saarlandes, PharmaScienceHub (PSH), 66123 Saarbrücken, Germany; 2 Medicinal Chemistry, Boehringer Ingelheim Pharma GmbH & Co KG, Birkendorfer Str. 65, 88397 Biberach an der Riss, Germany; 3 Open Molecular Software Foundation, Open Free Energy, Davis, CA 95616 USA

**Keywords:** Structure-based drug design, Chemical space exploration, Open-source drug design tools, Ligand optimization, SARS-CoV-2 inhibitors, Fragment-based drug design, Docking and scoring, Molecular dynamics simulations, Binding free energy calculations

## Abstract

We present Moldrug, a computational tool for accelerating the hit-to-lead phase in structure-based drug design. Moldrug explores the chemical space using structural modifications suggested by the CReM library and by optimizing an adaptable fitness function with a genetic algorithm. Moldrug is complemented by Moldrug-Dashboard, a cross-platform and user-friendly graphical interface tailored for the analysis of Moldrug simulations. To illustrate Moldrug, we designed new potential inhibitors targeting the main protease (M^Pro^) of SARS-CoV-2 by optimizing a consensus fitness function that balances binding affinity, drug-likeness, and synthetic accessibility. The designed molecules exhibited high chemical diversity. A subset of the designed molecules were ranked using MM/GBSA and alchemical binding free energy calculations, revealing predicted affinities as low as $-10\,~\hbox {kcal}\,\hbox {mol}^{-1}$. Moldrug is distributed as a Python package under the Apache 2.0 license. It offers pre-configured multi-parameter fitness functions for molecular design, while being highly adaptable for integrating functionalities from external software. Documentation and tutorials are available at https://moldrug.rtfd.io.

**Supplementary Information:**

The online version contains supplementary material available at 10.1186/s13321-025-01022-3.

## Scientific contribution

Moldrug is a modular and flexible open-source framework for efficient exploration of the chemical space without need of prior training. We demonstrated the applicability of Moldrug by designing new potential inhibitors for the M^Pro^ of SARS-CoV-2 with high predicted affinity according to alchemical free energy calculations. Moldrug follows good coding practice and is accompanied by detailed documentation, making Moldrug an accessible and adaptable resource for cheminformatics research.

**Graphical Abstract:**

**Supplementary Information:**

The online version contains supplementary material available at 10.1186/s13321-025-01022-3.

## Graphical abstract

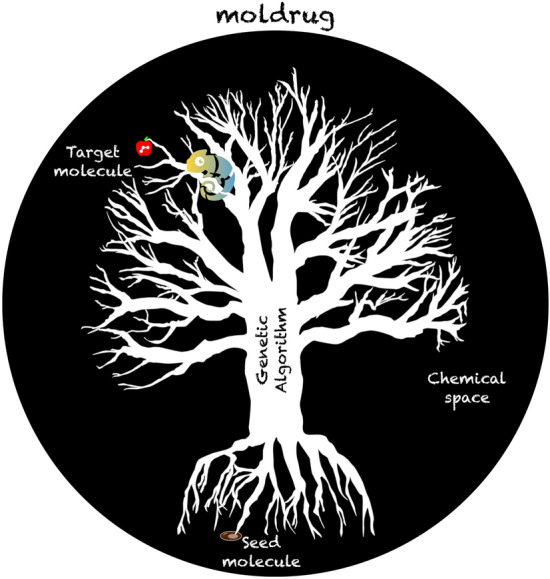

## Introduction

The hit-to-lead phase in drug design involves optimizing molecules to enhance their potency towards the desired target protein and improve their pharmacokinetic properties. Computational methods have proven to be invaluable in this regard, providing substantial support throughout the process [1]. Virtual screening plays a crucial role in identifying potential hit molecules and facilitating hit-to-lead generation [1]. Nowadays, virtual screening campaigns can screen from millions to billions of compounds [2]. Unfortunately, many of these hits bear structural similarity to known entities, making the creation of novel drugs conditional on lead optimization. This limitation reduces true novelty in drug design; however, some works have proposed workaround solutions [3].

A family of methods known as fragment-based drug design created high expectations in the early 1990s because they proposed a new approach to computational drug design without sacrificing chemical novelty. The idea was to use fragments of molecules as building blocks and link those to generate new molecules. Many software implementations follow this philosophy [4–11], with LUDI [4, 5] being one of the first. LUDI successfully optimized the potency of inhibitors targeting HIV protease and dihydrofolate reductase [5].

However, some challenges persisted. Many of these methods relied on the hypothesis that the core or seed molecule remains unchanged upon the addition of a new fragment, which is not always true [12]. Docking scoring functions, used to predict the binding affinity of the molecules, were in the development stages at the time. Available databases of fragments were still insufficient to explore the vast chemical space. All the methods were mainly focused on optimizing only the potency of the molecule. Designed molecules could request challenging synthetic routes. Lastly, designed molecules did not always exhibit drug-like properties.

A new generation of software and methodologies concentrated efforts on solving some of the known challenges by incorporating better docking algorithms, addressing the lack of drug-likeness and synthetic accessibility, improving the quality of the database of compounds or fragments and generalizing the use of evolutionary algorithms for the optimization process

[13–18]. The software AutoGrow [14] uses for the first time as a selection operator the scoring function of AutoDock [19]. OpenGrowth incorporates the concept of biasing the generation of molecules to statistically resemble drugs in an input training database to tackle the need for chemical realism in the proposed solutions [15]. Even attempts to fully enumerate subdomains of the “small molecule universe” (the set of all synthetically feasible organic molecules of $\approx 500 \hbox {Da}$) were carried out [17, 18].

The excitement surrounding artificial intelligence/machine learning (AI/ML) methodologies was also reflected in the field of de novo drug design [20–25]. However, data quality, training times, range of applicability, extrapolation, and achieving drug-likeness for the solutions remain topics under investigation.

Over the past four years, the field of de novo design has witnessed substantial advancements, marked by the introduction of novel software and methodologies in both fragment-based optimization and AI/ML strategies [26–38]. Additionally, technical progress, such as the integration of parallelization into AutoDock at both CPU and GPU levels [39] and the expanded integration capabilities of AutoDock-Vina [40], has significantly contributed to this forward momentum.

Noteworthy advances have emerged over the past four years. LigBuilder V3, introduces capabilities for multi-biologic-target optimization [29]. MolFinder employs an evolutionary algorithm that operates on the SMILES (Simplified Molecular-Input Line-Entry System) representation of molecules and employs fitness functions encompassing various properties like Quantitative Estimation of Drug (qed) likeness and Synthetic Accessibility (sa) score [34]. While MolFinder has exhibited promising results compared to state-of-the-art methodologies, its efficacy in designing molecules constrained by a biological target remains untested. Prentis et al. [35] demonstrate that the elitism selection method yields more tightly clustered molecules in terms of 2D/3D similarity, with more favorable fitness, followed by tournament and roulette selection methods. Fragment-based drug design methods have been synergistically combined with AI/ML approaches [31, 36]. Lu et al. introduces SECSE, a de novo design open-source software that primarily integrates rule-based molecular generation and structure-based drug design methods using genetic algorithms, along with on-the-fly deep learning training to mitigate the computational burden of docking evaluations [37].

During this period, Polishchuk introduced CReM [27, 28], an elegant and versatile open-source molecular generator. This method utilizes the concept of interchangeable fragments. For instance, if two molecules with distinct cores share identical peripheral fragments up to a predefined number of heavy atoms, then the cores of each molecule become interchangeable. This basic concept expands the chemical novelty with a native incorporation of chemical realism to the designed molecules. Moreover, the adaptable implementation of CReM may position it as a reference Python library in the future of de novo drug design. With CReM, it is possible to select specific local chemical features of the molecule to be optimized, rather than treating the entire molecule as a single entity, as many past methods have done. During the preparation of this manuscript Minibaeva and Polishchuk [41] combined CReM with docking algorithms, yielding promising results.

Here, we present Moldrug, an efficient and versatile open-source chemical space explorer. Moldrug leverages the flexible CReM framework [27, 28] as its structure generator, expanding upon its functionalities for an iterative environment. CReM equips Moldrug with a highly adaptable mechanism to traverse the chemical landscape, offering fine user control over exploration strategies and chemical novelty while maintaining chemical realism.

Molecules are guided with an evolutionary algorithm reminiscent of a classical genetic algorithm [42], albeit without crossover operations. Moldrug facilitates multi-property-oriented optimization through the utilization of Derringer-Suich desirability functions [43], a less-explored concept in de novo drug design literature. Variables such as sa_score (synthetic accessibility score) or qed (Quantitative Estimation of Drug-likeness) are no longer static filters during optimization; in Moldrug, they actively guide the optimization process.

Moldrug ships with robust fitness functions tailored for drug design, capable of performing constrained docking, multi-biological-target optimization, and other features discussed in detail below. However, Moldrug is not confined to predefined evaluation strategies; it allows users to seamlessly define custom fitness functions to better suit their specific requirements. This flexibility theoretically renders Moldrug suitable for applications beyond the scope of drug design.

In this study, we also explored the combination of the Moldrug algorithm with molecular dynamics simulations as a post-processing step, focusing on pose stability prediction, interaction profiles, and alchemical binding free energy calculations. This combination, referred to as “mouse”, demonstrated significant value in refining and validating proposed solutions. The post-processing steps highlighted the efficiency of Moldrug and underscored the importance of molecular dynamics analysis in the final selection of putative drugs for subsequent wet lab testing.

Moldrug is implemented in Python and takes the form of a modular program, allowing for extensive customization and integration into diverse drug discovery pipelines. Its user interface has been meticulously designed for simplicity, reducing the learning curve for researchers. Moldrug is complemented by Moldrug-Dashboard, a cross-platform and user-friendly graphical interface tailored for the analysis of Moldrug simulations.

Moreover, Moldrug is committed to user-centric development. We continuously update its documentation, augmenting it with tutorials and incorporating new features to enhance user experience. Our tutorials are designed to be executable either locally or online through platforms like Binder or Google Colab, and the relevant links are provided in our online documentation. Moldrug is open-source software released under the Apache 2.0 license, ensuring accessibility and fostering collaborative research. The online documentation is available at: https://moldrug.rtfd.io.

## Implementation

**Fig. 1:**
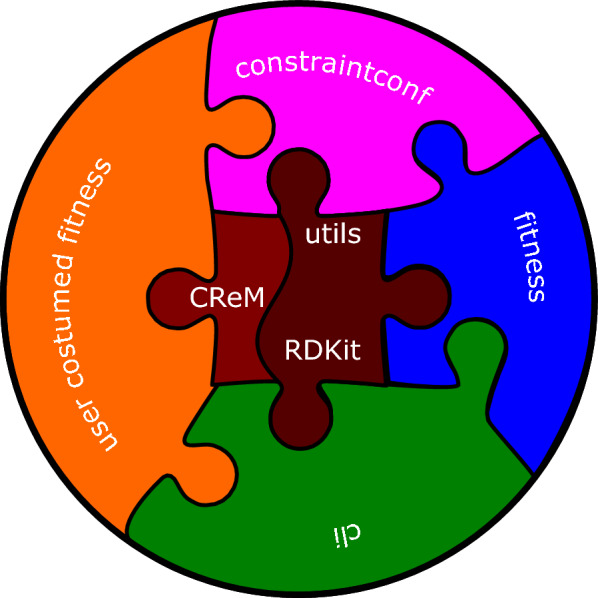
Schematic representation of Moldrug’s modules

Moldrug is a Python software featuring a modular architecture showcased in Fig. 1. At its core lies the *utils* module, home to pivotal classes like: *Individual*, *Local* and *GA*, and different functions and classes that collectively form the backbone of Moldrug’s capabilities. The *utils* module is complemented with a Command Line Interface (*cli*), capabilities to perform constrained docking (*constraintconf*) and four build-in fitness functions: *Cost*, *CostOnlyVina*, *CostMultiReceptors* and *CostMultiReceptorsOnlyVina* (within the *fitness* module). In addition, Moldrug stands out by integrating user-customized fitness functions, enhancing adaptability and user-friendly operation.

### The *Individual* class

An *Individual* object serves as the primary input for any fitness function, whether built-in or user-customized (refer to section *Fitness Function* for details on fitness functions). The *Individual* class encompasses five key attributes:

1. *mol*: Represents the RDKit [44] molecule object.
2. *idx*: Identifies the *Individual*.
3. *pdbqt*: A pdbqt string representation of the molecule, generated through the Meeko [45] Python library. This pdbqt string is compatible with AutoDock-Vina [46].
4. *smiles*: Denotes the SMILES (Simplified Molecular-Input Line-Entry System) of the molecule without explicit hydrogens. Set as an immutable property, *smiles* serves as *hash* and ensures equality between instances. Two *Individual* instances are considered to be equal (with operation *==*) only if they share the same *smiles* property.
5. *cost*: A *float* number representing the fitness of the *Individual*. This value must be updated during the evaluation of the fitness function. Arithmetic operations between instances of *Individual* use this attribute.

### Fitness function

**Fig. 2:**
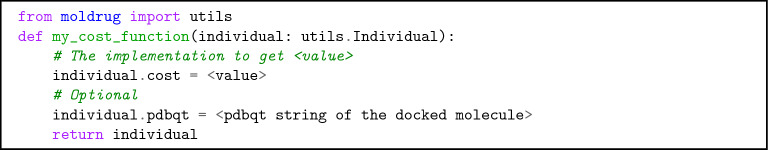
Fitness function template compatible with *GA* and *Local* classes

Moldrug can theoretically accommodate any fitness function, subject to the following constraints:

- The fitness function must be a serializable object for two key reasons: (**i**) The *GA* and *Local* classes internally use the Python *Multiprocessing* module during the evaluation phase, where functions need to be passed between different processes, which requires serialization. (**ii**) The fitness function is also stored as an attribute of the *GA* and *Local* classes, and these classes can be saved to disk using the *pickle* or *dill* Python modules, both of which require objects to be serializable in order to save and load them. Since most Python objects are serializable, this requirement only poses minor limitations.
- The first argument of the fitness function must be an *Individual* object, and it should return the modified *Individual* with the updated *cost* attribute, where a lower value indicates a better fit. The structure of a fitness function is illustrated in Fig. 2. The user may optionally update the *pdbqt* attribute with a PDBQT string of the docked conformation. This attribute is used by the function *make_sdf* of the *utils* module to create an SDF file with the conformations of a list of *Individual*’s.
- Since the parallelization is implemented with the *Multiprocessing* Python module that utilizes a shared-memory scheme. The fitness function should not be excessively computationally expensive and should not require excessive use of memory.

In the initial stages of drug design, the primary focus is typically on identifying molecules with a robust binding affinity to the target biomolecule. Subsequent steps involve refining the drug-likeness profile. While this conventional approach has demonstrated success, the intricate task of finely adjusting the drug-likeness properties, while preserving or enhancing the achieved potency, may be challenging.

A drug should simultaneously fulfill several properties. For our built-in fitness functions *Cost* and *CostMultiReceptors*, we describe the drug with AutoDock-Vina Score [46] (*vina_score*), Quantitative Estimation of Drug-likeness [47] (*qed*) and the Synthetic Accessibility Score [48] (*sa_score*). *vina_score* accounts for the potency of the molecule against the bimolecular target; *qed* is a measure of the drug-likeness and *sa_score* estimates ease of synthesis.

#### Desirability functions

Each property is scaled using Derringer-Suich desirability functions [43]. In brief, the desirability function transforms each property to a desirability value $d_i$, where $0 \le d_i \le 1$ and $d_i = 1$ represents the optimal value. Then, all $d_i$ values are combined using a weighted geometric mean; where $w_i$ is the weight of desirability $d_i$:

1 $$\begin{aligned} D = \left( \prod _{i=1}^{N} {d_i}^{w_i} \right) ^{1 / {\sum _{i=1}^{N} w_i}} ~\text {;where}~ 0 \le D \le 1; \end{aligned}$$

Moldrug incorporates three desirability functions, accessible within the *utils* module: *LargerTheBest* (Eq. 2), *SmallerTheBest* (Eq. 3), and *NominalTheBest* (Eq. 4). These functions are designed for situations where optimization aims for a maximum, a minimum, or falls within a specified range for a particular property, respectively.

2 $$\begin{aligned} & {\texttt {\textit{LargerTheBest}}} = {\left\{ \begin{array}{ll} 0 & , x \in (-\infty ; l) \\ {\left[ \frac{x - l}{t - l} \right] }^{r} & ,x \in [l; t] \\ 1 & , x \in (t; \infty ) \end{array}\right. } \end{aligned}$$

3 $$\begin{aligned} & {\texttt {\textit{SmallerTheBest}}} = {\left\{ \begin{array}{ll} 1 & , x \in (-\infty ; t) \\ {\left[ \frac{u - x}{u - t} \right] }^{r} & ,x \in [t; u] \\ 0 & , x \in (u; \infty ) \end{array}\right. } \end{aligned}$$

4 $$\begin{aligned} & {\texttt {\textit{NominalTheBest}}} = {\left\{ \begin{array}{ll} 0 & , x \in (-\infty ; l) \\ {\left[ \frac{x - l}{t - l} \right] }^{r_1} & ,x \in [l; t] \\ {\left[ \frac{u - x}{u - t} \right] }^{r_2} & ,x \in (t; u] \\ 0 & , x \in (u; \infty ) \end{array}\right. } \end{aligned}$$

Where *x*, *l*, *u*, and *t* correspond to the parameters *Value*, *LowerLimit*, *UpperLimit*, and *Target*, respectively, in the code. These parameters are used to define the range and thresholds for the desirability functions:

- *Value* (*x*): the property value being evaluated.
- *LowerLimit* (*l*): the minimum acceptable value for the property.
- *UpperLimit* (*u*): the maximum acceptable value for the property.
- *Target* (*t*): the optimal value that the property should reach or approach.

The parameters *r*, $r_1$, and $r_2$ control the steepness of the transitions in the desirability functions.

Depending on the properties targeted for optimization, users have the flexibility to choose specific desirability functions and fine-tune their contribution to the weighted mean by adjusting $w_i$ in Eq. 1 and the values of *r* in Eqs. 2 and 3, as well as $r_1$ and $r_2$ in Eq. 4. The relationship between desirabilities and the parameter *r* is illustrated in Fig. S1. The use of desirability functions offers high flexibility, giving control over the exploration of the chemical space according to specific needs.

It is, however, crucial to acknowledge that with an increase in the number of properties incorporated into the fitness function through a desirability function, the complexity of the optimization may proportionally escalate.

#### Built-in fitnesss functions

Moldrug’s *fitness* module includes four pre-implemented fitness functions, each designed for specific scenarios. These functions update the *cost* attribute of the provided *Individual* with a floating-point number, with a lower value indicating a better fit.

The *Cost* fitness function updates the *cost* of the *Individual* with $1-D$ where *D* is the weighted geometric mean of the desirability values *vina_score*, *qed* and *sa_score* (See Eqs. 1, 2, 3 and 4). For certain applications, users may focus solely on optimizing the molecule’s potency. While *Cost* can serve this purpose by adjusting the desirability functions of *qed* and *sa_score*, the fitness function *CostOnlyVina* is specifically designed for this scenario. It updates the *cost* with the *vina_score* value only.

*CostMultiReceptors* and *CostMultiReceptorsOnlyVina*, generalize the previous fitness functions to accommodate multiple receptors. These functions enable the incorporation of potency information across several receptors; particularity useful when the drug should be selected for binding to one protein over another. In scenarios involving flexible receptors, ensemble docking [49] can be performed using these functions. Software like LigBuilder V3 [29] and MolPAL [50] also include multi-biological-target capabilities using different approaches.

The Moldrug GitHub repository hosts additional fitness function examples in the Contrib directory that are not included in the main Python distribution but are fully accessible and compatible with Moldrug. At the time of writing this paper, two projects are available: one utilizes MolSkill [51] as a drug-likeness predictor instead of *qed*, while the other trains and employs machine learning models for the prediction of specific ADMET properties. We envision this directory to host various fitness functions built by and for the community in the future.

### The *GA* (Genetic algorithm) class

**Fig. 3:**
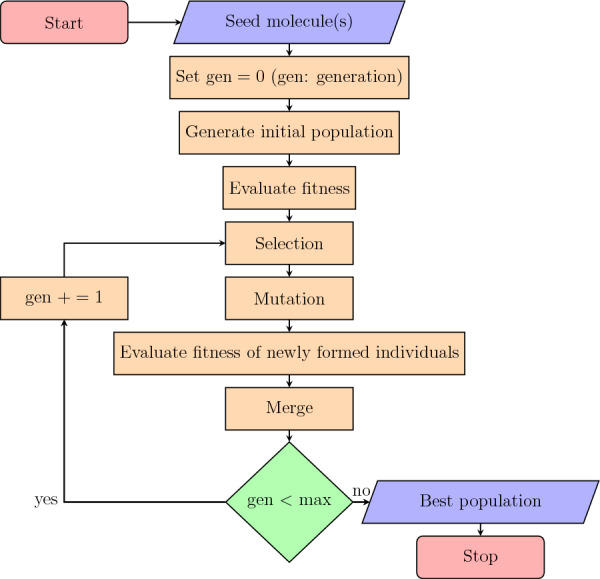
Optimization flowchart followed by the *GA* class

*GA* is the main Moldrug class. It carries out the optimization of the input chemical structure(s). It follows the structure of a classical genetic algorithm [42] but excludes the crossover operation that proved being inefficient for our application case. The *GA* class comes with various attributes and methods, as detailed in the online documentation.

*GA* is a callable object, implying that the *__call__* method is implemented. This method initializes the population with a size specified by the attribute *popsize*, based on the attributes *_seed_mol* and *mutate_crem_kwargs*. Figure 3 illustrates the optimization flowchart followed by the *GA* class. Following population initialization, individuals undergo evaluation based on user-provided fitness functions. The *costfunc* and *costfunc_kwargs* attributes store the fitness function object and its corresponding arguments, respectively. As initial molecule(s) defined by *_seed_mol*, either an identified hit molecule or the simple methane molecule may be used.

Individuals are then selected for further mutations using a roulette wheel selection strategy [42] with constant selection pressure. The probability of choosing an individual is calculated based on a Boltzmann distribution. Where $\beta$ represents the constant selection pressure:

5 $$\begin{aligned} P_i = \frac{e^{-\beta \textrm{cost}_i}}{\sum _{i=1}^{\textrm{popsize}} e^{-\beta \textrm{cost}_i}} \end{aligned}$$

A large $\beta$ value increases the likelihood of selecting the best-fit individual, while a small $\beta$ value leads to a more equal distribution of probabilities, fostering a more exploratory selection process. The $\beta$ parameter is defined as the attribute *beta* in the *GA* class. In future versions of Moldrug, additional selection strategies such as Rank Selection, Tournament Selection, Boltzmann Selection, or Stochastic Universal Sampling [52] may be considered. However, our current selection strategy has proven to be efficient.

Selected individuals are submitted to mutation through the CReM [27] Python library. CReM is a fragment-based structure generation that excels in its customization and flexibility while exploring the chemical space. The parameters for CReM are accessed through the *mutate_crem_kwargs* attribute of the *GA* class. CReM mutations can be conceptualized as an external crossover, introducing fragments from a database of interchangeable fragments into the current population, and eliminating the need for an internal crossover in our method. CreM databases of interchangeable fragments can be downloaded at www.qsar4u.com or built from scratch by the user.

The offspring resulting from the CReM mutations are then evaluated based on the fitness function. To maintain a constant population size, the previous and offspring populations are merged, and individuals with the highest *cost* are discarded.

If the maximum number of generations (attribute *maxiter*) is reached, the simulation stops. If not, the selection, mutation, evaluation and merge steps are repeated.

The *GA* class can be invoked multiple times. Across different calls, the attributes of the *GA* class can be modified to choose a different search strategy. The *GA* class maintains an internal state through its attributes, so multiple invocations of the class can continue from where the previous call left off, effectively allowing for a continuous simulation rather than resetting each time. This flexibility enables for the implementation of different search strategies within the same simulation. Furthermore, the class is a serializable object, suggesting that it can be saved to disk for future use, currently using the *pickle* method.

**Fig. 4:**
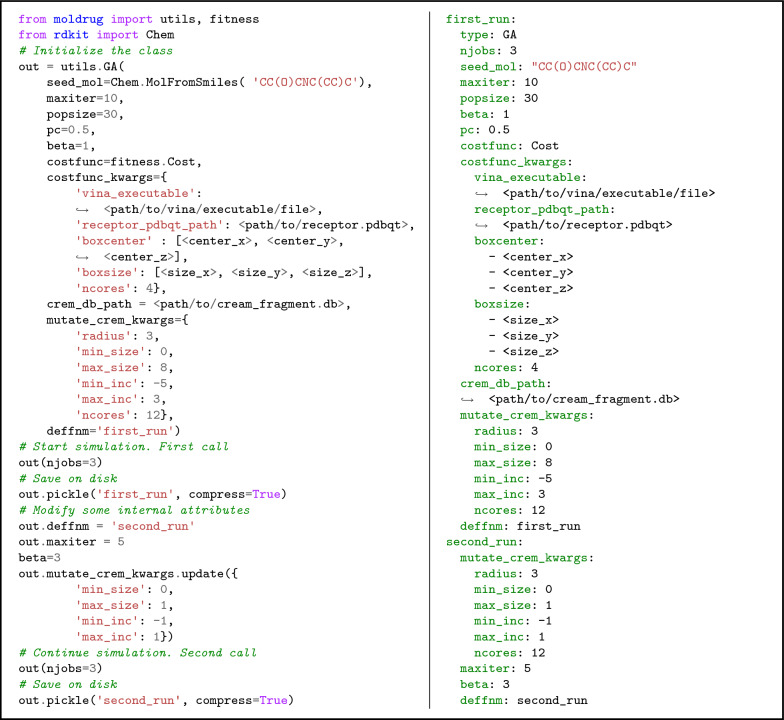
Example of the utilization of the *GA* class in Moldrug. The example includes initialization, a first run and saving to disk, modification of internal attributes, a second run and final saving to disk. **Left**: Python code snippet, **Right**: configuration file for the command line interface. Only *seed_mol*, *costfunc*, *costfunc_kwargs* and *crem_db_path* are mandatory parameters during the initialization of the class

To perform a *GA* optimization in Moldrug, users can initialize the *GA* class with minimal mandatory parameters such as *seed_mol*, *costfunc*, *costfunc_kwargs*, and *crem_db_path*. For a more advanced use case, Fig. 4 illustrates a Python code snippet. The example initializes the class, specifying the molecule to be optimized (*seed_mol*) over 10 generations (*maxiter*) with a population size of 30 individuals (*popsize*), a proportion of children of 0.5 (*pc*) and selection pressure of 1 (*beta*). The fitness function *Cost* is employed (*costfunc*), along with its specified parameters (*costfunc_kwargs*). The path to the CreM database (*crem_db_path*) and parameters for the *mutate* CReM function (*mutate_crem_kwargs*) are set accordingly. A default prefix name is given (*deffnm*) for output files. The class is then called with a parallelization level of three evaluations per generation (*njobs*). In the subsequent run, lasting for an additional 5 generations, only atom-per-atom substitution is allowed and the selection pressure is increased to 3. The results of each run are saved with distinctive names using the *pickle* method. More advanced options and functionalities are detailed in the online documentation.

### The *Local* class

*Local* is an auxiliary class of Moldrug, not designed for independent optimization. However, in the later phases of drug discovery or lead optimization, this functionality proves particularly beneficial. The *Local* class may be considered as a wrapper around the *grow_mol* function of CReM but enhanced with attributes and methods for performing evaluations (*__call__*) of a provided fitness function, saving to disk (*pickle*), and retrieving data (*to_dataframe*).

Similar to *GA*, *Local* is a callable object. The *__call__* method grows the seed molecule specified by the attribute *_seed_mol* with the parameters of the attribute *grow_crem_kwargs*. Figure S2 illustrates the simple workflow of the *Local* class. After the growing step, a number of offspring are selected (or picked) based on the *pick* parameter of the *__call__* method. Finally, the selected offsprings are evaluated with the fitness functions provided by the user. The *costfunc* and *costfunc_kwargs* attributes store the fitness function object and its corresponding arguments, respectively. The left side of Fig. S3 shows an example of how to use the *Local* class inside a Python code.

### Command Line Interface (CLI)

The Moldrug package, primarily designed for usage as a Python module, also provides a command line interface (CLI) covering most of its standard functionalities. *moldrug* is the main command and requires a positional argument-the configuration YAML file. This file outlines simulation parameters similarly to using Moldrug as a Python module. Custom fitness modules can be incorporated by providing the path to the Python file using the *–f* or *–fitness* flag. Additionally, the *–c* or *–continue* flag allows for the continuation of a simulation. Examples of configuration files are presented in Figs. 4 and S3 (right column), along with their corresponding Python code counterparts (left column).

### Constrained docking: the *constraintconf* module

This module was developed as part of a community effort, initially introduced by Dudgeon [53] and later improved by Meyers [54] and Walters [55]. Our contributions include the introduction of a new command line interface, enhancements in conformer generation for scenarios where previous solutions fall short, handling of specific exceptions, modification of clashing conformation detection and the incorporation of additional documentation.

The approach involves generating new embedded or, if the embedding strategy fails, aligned conformations for a specified molecule relative to a reference. Subsequently, conformations that collide with the protein are removed.

The *constraintconf* module is integrated into all built-in Moldrug fitness functions enabling constrained docking. The resultant conformation can be evaluated using a docking engine such as AutoDock-Vina [46], obviating the conformation exploration and restricting to evaluation. Built-in Moldrug fitness functions support both local optimization and score-only capabilities of AutoDock-Vina through the keyword *constraint_type*, which accepts either “*local_only*” for local optimization or “*score_only*” for score-only evaluation. During local optimization the constrained conformation is locally relaxed by AutoDock-Vina while score-only return the docking score of the proposed constrained conformation without any further optimization.

Constrained docking is powerful in scenarios where the preservation of not only the chemical structure of the seed molecule (achieved through CReM parameters) but also its three-dimensional conformation is crucial. This feature is particularly valuable in the advanced stages of drug discovery or lead optimization.

**Fig. 5:**
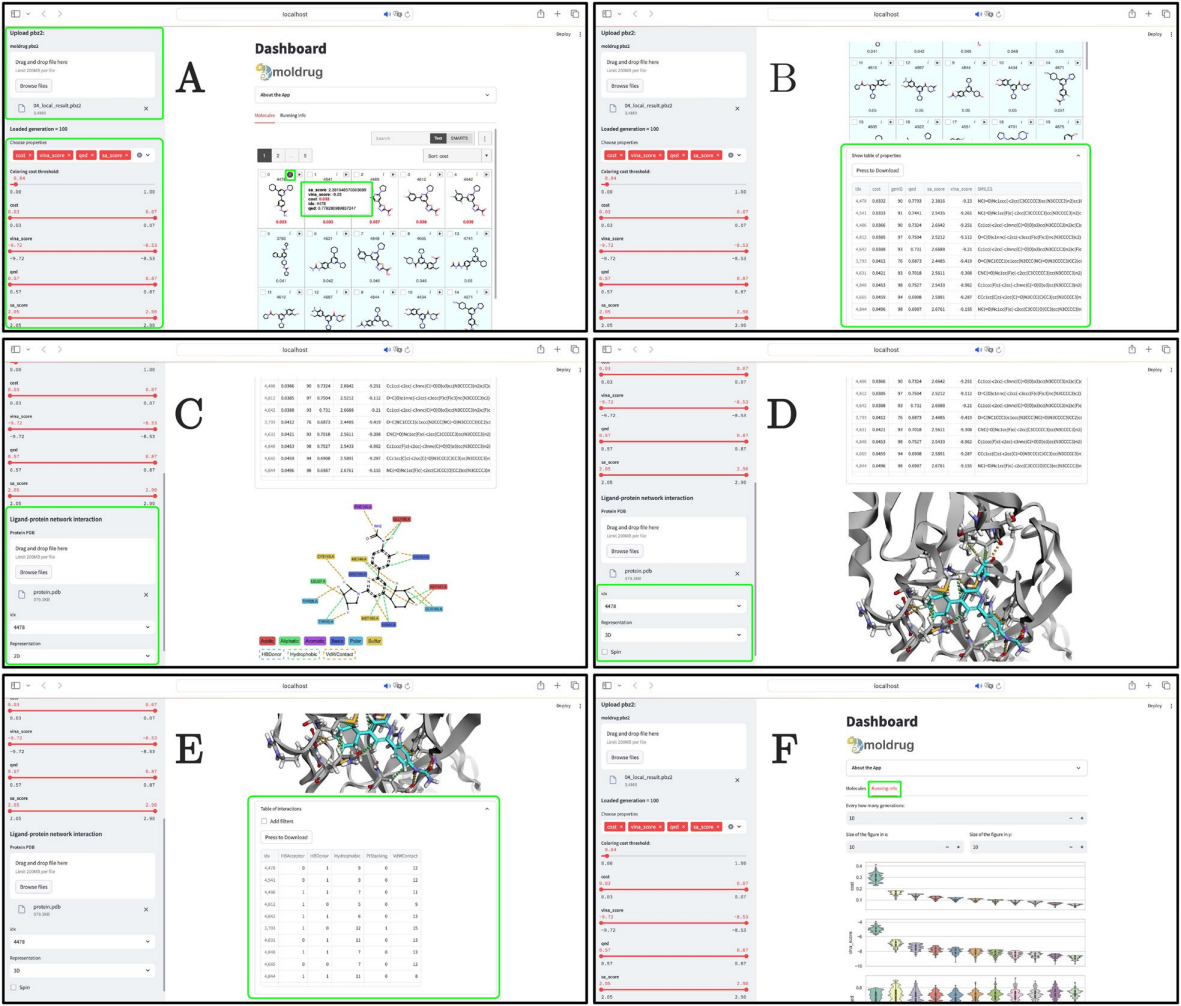
Snapshots of Moldrug-Dashboard. Green boxes highlight key features. See the main text for more details. The illustrated example corresponds to the free campaign presented in this document

### Moldrug-dashboard: an interactive analysis tool

Moldrug-Dashboard is a Streamlit application designed for the interactive analysis of Moldrug results. The application’s capabilities are made possible through the integration of various open-source projects such as mols2 grid [56] and ProLIF [57]. The complete list of dependencies is listed in the Moldrug’s GitHub repository at streamlit/requirements.txt. Moldrug-Dashboard is accessible online at https://moldrug-dashboard.streamlit.app or can be run locally using the script moldrug-dashboard.py, available in the Moldrug’s GitHub repository. The online Moldrug’s documentation provides detailed instructions for installation and usage.

To utilize Moldrug-Dashboard, a mandatory file is required: a compressed serialized object of either the *Local* or *GA* classes, or a tuple with an integer as its first element and a list of *Individual*’s as its second element. This file, with the extension *.pbz2*, is automatically generated upon executing the *moldrug* command. Alternatively, it can be generated using the *pickle* method of the *Local* or *GA* classes or with the *compressed_pickle* function from the *utils* module with the keyword *compress = True*. Additionally, the optional inclusion of a PDB file of the protein in the dashboard is recommended if docking was performed, and the *pdbqt* attribute of the *Individual* was properly updated during evaluation (see section *Fitness Function* for more details about it).

Moldrug-Dashboard facilitates interactive analysis, allowing users to dynamically filter results based on selected optimization properties. The left sidebar (Fig. 5 A) features sliders for each property, influencing the entire dashboard. The chemical structures table, generated using mols2grid software [56], is fully interactive. A summary table of properties, sortable and downloadable, is also accessible (Fig. 5 B).

If a protein is loaded in Moldrug-Dashboard, the ProLIF software [57] calculates the protein-ligand interaction fingerprint. Two interactive representations, either 2D (Fig. 5 C) or 3D (Fig. 5 D), are available for a specified solution. A comprehensive table of interaction types for all solutions is provided, enabling sorting, filtering, and download functionalities (Fig. 5 E).

To analyze the optimization process, Moldrug-Dashboard offers a view of the population’s evolution across generations (Fig. 5 F). This feature is pivotal in assessing the effectiveness of the employed optimization strategy.

## Methods

### Structure Retrieving and processing

The Moldrug methodology is here presented for its applicability in designing new inhibitors targeting the main protease (M^Pro^) of SARS-CoV-2, as crystallized by Jin et al. [58] in complex with the N3 inhibitor (PDB code: 6LU7). The protein and ligand were separated. Hydrogen atoms were added to the protein (pH = 7) and missing atom corrected using the PDB2PQR program [59]. Subsequently, the PDBQT file of the protein was generated using the prepare_receptor script from ADFRsuite-1.0 [60]. The ligand was processed with RDKit [44] and missing valances were added manually.

### Selection of case examples

During the initial stages of drug design, binding information is usually available for fragments, often lacking clear insights into binding poses. To illustrate Moldrug’s utility in this challenging context, we chose the 2-phenylethanol fragment of the N3 inhibitor as the seed molecule (see Fig. S4). This selected fragment, not extensively detailed in the experiment (Fig. S5), introduces ambiguity, similar to situations in early-stage drug design projects characterized by significant uncertainties in initial system knowledge.

Here, we present two Moldrug case studies: *free campaign*, wherein AutoDock-Vina optimizes the docking pose without restrictions on the generated putative inhibitors, and *constrained campaign*, employing Moldrug’s *constraintconf* module for docking with the selected seed molecule as tridimensional conformation reference and the heavy atoms of the seed molecule remaining unchanged across all generations in terms of both chemical structure and tridimensional conformation.

### Moldrug parameters

The 3.7.2 version of Moldrug was employed. The docking box, centered at (−8.5, 16.5, 67.5) Å with dimensions 23.0 (X), 35.0 (Y), and 33.0 Å (Z), encloses the cavity with the N3 inhibitor. Although larger than necessary, this size avoids biasing inhibitor design based on N3 binding mode. This deliberate box selection aligns with our goal to emulate the typical uncertain conditions of early-stage drug design projects. The exhaustiveness parameter of AutoDock-Vina was set to 9.

The Moldrug optimization process comprised 100 generations, divided into four stages. Each stage involved a population size (*popsize*) of 100 individuals, a proportion of children (*pc*) of 0.5, selection pressure (*beta*) of 1 and CReM database of interchangeable fragments with maximum synthetic accessibility score of 2 [27, 28]. The *Cost* fitness function was used with desirability values listed in Table 1.

**Table 1:** Desirability values used by the *Cost* fitness function

**Property**	**Function**	**LowerLimit**	**UpperLimit**	**Target**	**r**	**w**
*vina_score*	*SmallerTheBest*	-	$-2$	$-10$	1	1
*qed*	*LargerTheBest*	0.1	-	0.75	1	1
*sa_score*	*SmallerTheBest*	-	7	3	1	1

The optimization stages are shown in Table 2 along with the corresponding CReM parameters. The initial 20 generations focused on mutating only hydrogen atoms by fragments with a number of heavy atoms between 1 and 6 (*01_grow*). Subsequently, 25 generations involved the mutation of hydrogen atoms and up to two heavy atoms by fragments with heavy atoms ranging from $-2$ (deletion of heavy atoms in the mutated molecule) to 4 (*02_allow_grow*). This is followed by 40 generations where only up to 8 heavy atoms were mutated, using fragments with heavy atoms between $-5$ (deletion of heavy atoms in the mutated molecule) and 3 (*03_pure_mutate*). Finally, 15 generations performed point mutations, replacing, deleting, or maintaining one heavy atom (*04_local*). These sequential stages are inspired by the progression from lead generation to lead optimization. In the constrained campaign, all heavy atoms of the seed molecule were safeguarded against mutation throughout all generations.

**Table 2:** Parameters passed by Moldrug to the CReM *mutate_mol* function and the number of generations spend on each step

**Stage**	**radius**	**min_size**	**max_size**	**min_inc**	**max_inc**	**generations**
01_grow	3	0	0	1	6	20
02_allow_grow	3	0	2	$-2$	4	25
03_pure_mutate	3	1	8	$-5$	3	40
04_local	3	0	1	$-1$	1	15

### Similarity analysis

Molecular fingerprint was calculated with a Morgan fingerprint of radius 2 and 2048 bits as implemented in the RDKit Python library [44].

#### Inter- and Intra-campaign similarities

To compute the pair-wise similarity among the designed molecules of each campaign, the Tanimoto similarity was computed on the molecular fingerprints. Tanimoto similarity ranges from 0 (no similarity) to 1 (identical molecules). Histograms of the distribution were created with 300 bins, and kernel density estimation (KDE) was applied to provide a smoother representation of the underlying distribution. This was done using the Seaborn Python library [61].

Two types of analysis were conducted: **i** the intra-campaign similarity analysis, where the similarity among the molecules within the same campaign was calculated, and **ii** the inter-campaign similarity analysis, where each molecule from one campaign was compared to all the molecules from the other campaign.

The intra-campaign similarity measures how thoroughly a single simulation explores the chemical space, while the inter-campaign similarity evaluates the exploration of distinct regions of the chemical space when different strategies are employed. Together, both analyses provide insights into whether Moldrug is capable of designing novel molecules and avoiding being trapped in local minima of the chemical space.

#### Quantification of chemical space coverage

To quantify the chemical space explored in each campaign, we projected the designed molecules onto the Enamine-Hit Locator Library (HLL-460), which contains 460,160 diverse and synthesizable compounds. The chemical space was constructed based on the molecular fingerprints for both the Enamine compounds and the molecules designed during the free and constrained Moldrug campaigns, including both accepted and rejected candidates. We first performed Principal Component Analysis (PCA) to reduce the dimensionality of the fingerprint data, keeping the top 50 principal components (PCs). Then, the t-distributed Stochastic Neighbor Embedding (t-SNE) was applied to these PCs to further reduce dimensionality to two components for visualization. The percent of covered variance by the 50 first PCs was computed and reported. All PCA and t-SNE analyses were performed using the scikit-learn Python package [62].

### Binding affinity calculations by molecular dynamic simulations

#### Molecular mechanic generalized Born surface area (MM/GBSA) calculations

The molecules from the last population of each stage were submitted for MM/GBSA calculations. AMBER99SB-ILDN force field was used for protein parameters in GROMACS 2021 [63]; ligand parameters and partial charges were assigned using the Open Force field (v2.1.0) [64] and the AM1-BCC partial charge model respectively. The protein-ligand complexes were solvated in TIP3P [65] water model in octahedron boxes with a minimum distance of 12 Å from the solute to the box edge using GROMACS modules. Sodium and chloride ions were added to neutralize the systems and achieve a concentration of 150 mM. ParmEd version 3.2.0 (https://github.com/ParmEd/ParmEd) was used to convert input topologies and coordinates between Open Force Field and GROMACS file formats. Hydrogen mass repartitioning (HMR) [66, 67] was used to achieve a 4 fs integration time step for all simulations; hydrogen masses, except those of waters, were increased to 3 amu by redistributing the mass from the corresponding linked heavy atoms. Hydrogen motions were constrained using LINCS [68, 69] algorithm. In all cases, simulation temperature was maintained at 298.15 K. A simulation pressure of 1 atmosphere was maintained using Berendsen barostat [70] during equilibration with a time constant of 1 ps, followed by the Parrinello-Rahman barostat [71] with a time constant of 2 ps for production simulations. A cut-off of 1 nm was used for short range interactions, and long range electrostatics were computed with the particle-mesh Ewald method [72, 73]. During this equilibration, protein backbone atoms and non-hydrogen ligand atoms were restrained using a $5 ~\hbox {kcal}/\hbox {mol}/{\text{\AA }}^{2}$ force constant. The simulation system was first minimized followed by the equilibration and the final frame from the equilibrated system was used to simulate 3 replicas of 50 ns each. The trajectory analysis was performed using GROMACS [63] tools and MDAnalysis package [74]. The approximate binding affinity, MM/GBSA, was calculated by protocol described by Maffucci et al. [75].

#### Alchemical Binding free energy calculations

From the top 100 molecules ranked by MM/GBSA, duplicates and molecules deemed impractical for relative binding free energy (RBFE) calculations were excluded. The remaining on each campaign were selected for RBFE calculations.

#### Relative binding free energy (RBFE) calculations

The relative free energy calculations were performed using the OpenFE 0.14 package. [76] The calculation networks were generated using an automated workflow build of the AtomMapper in Kartograf [77], the atom mapping scorer build from LOMAP [78], and the network building tool Konnektor [79] using the Starry Sky Network. First, all enumerated molecules were featurized with Morgan Fingerprints [80] and scikit-mol [81]. Next, the featurized molecules were clustered with the HDBSCAN implementation from scikit-learn [62, 82] and the provided default parameters. Members of each cluster, except the outlier molecules in the “noise”-cluster, were converted into star maps by finding all possible mappings inside the clusters with Kartograf’s atom mapper allowing a maximal distance of 0.95 Å and disallowing any ring size changes or breaking. The star map was created by placing the molecule with the highest average LOMAP atom mapping score at the center of each cluster. Clusters were concatenated by computing the bipartite matching for each pair of networks and then selecting the two highest scoring atom mappings from each pairwise matching. For the concatenation step, the same settings were used for Kartograf as before to find all possible mappings for the matching. The planned networks were executed utilizing the relative free energy protocol by OpenFE, which is based on the Perses toolkit implementation. [83] For the RFE-Protocol the parametrization of the ligands was performed with OpenFF 2.1.0 [64] and the protein was parametrized with AMBERFF14SB [84]. An integration time step of 4 fs was used because of the use of hydrogen mass repartitioning (HMR) scheme [66, 67]. The phase space of the system was sampled using Langevin integration [85] with a Hamiltonian Replica exchange approach for the $\lambda$ dimension. [86, 87] For more details please refer to [77] Each relative free energy transformation was attempted up to three times.

#### Absolute binding free energy (ABFE) calculations. Converting RBFE to ABFE

Absolute binding free energy (ABFE) calculations were performed for a representative molecule from each campaign, enabling the estimation of ABFE for the remaining molecules as follows:

6 $$\begin{aligned} \begin{aligned} \Delta G_{i}&= \Delta \Delta G_{i} + \Delta G_{r} - \Delta \Delta G_{r} \\ \delta (\Delta G_{i})&= \sqrt{\delta ^2(\Delta \Delta G_{i}) + \delta ^2(\Delta G_{r}) + \delta ^2(\Delta \Delta G_{r})} \end{aligned} \end{aligned}$$

Here, $\Delta \Delta$ denotes the relative binding free energy, $\Delta$ the absolute binding free energy, and $\delta$ the associated error. Subscripts *i* and *r* refer to the *i*-th molecule and the selected representative molecule for each campaign, respectively.

Additionally, ABFE was calculated for the N3 inhibitor from the crystal structure proposed by [88], allowing direct comparison.

All ABFE calculations were performed using the ABFE workflow described by Ries et al. [89], employing the same force fields used in the RBFE calculations.

## Results and discussion

### Performance and coverage of chemical space

Moldrug simulations were conducted on an $\textrm{AMD EPYC 7662}^{\textrm{TM}}$ 64-Core processor with 2 threads per core. The simulation took 7.5 hours for the free campaign and 8.0 hours for the constrained campaign, resulting in an overall performance close to 13 generations per hour across both campaigns. Throughout 100 generations, a total of 4956 and 4792 new molecules were generated and evaluated for the free and constrained campaigns, respectively. The constrained campaign generated only 164 fewer molecules than the free campaign, suggesting that the constraints had minimal impact on the efficiency of the molecule generation process. The acceptance rates for both campaigns were approximately 10 % (Fig. S16). The steady acceptance rate of around 10 % over 100 generations points to the robustness of the selection criteria within the genetic algorithm. This steady state indicates that the algorithm has reached a balance between exploration and exploitation, ensuring a consistent influx of viable molecule designs.

**Fig. 6:**
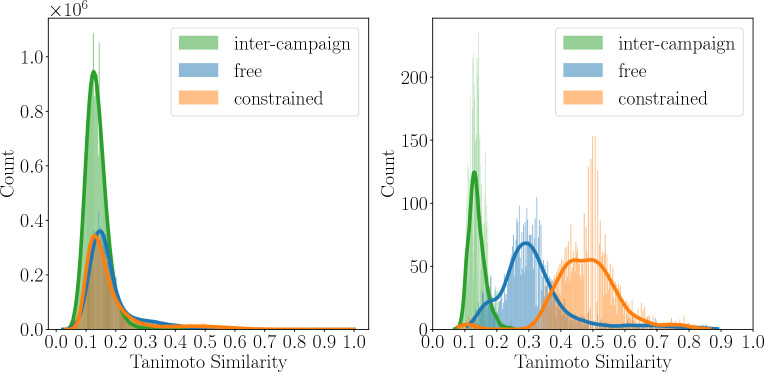
Pairwise similarity distribution among all designed molecules (**left**) and accepted molecules from the final generation (**right**) within each campaign (intra-campaign similarities) and between the two campaigns (inter-campaign similarities). Tanimoto similarity was calculated using the Morgan fingerprint with a radius of 2 and 2048 bits. For clarity, lines represent smoothed histograms, computed using kernel density estimation (KDE) as described in the methods section. Tanimoto similarity ranges from 0 (no similarity) to 1 (identical molecules)

To quantify the diversity of the designed molecules within each campaign, we calculated the pairwise intra-campaign Tanimoto similarity for all molecules (Fig. 6, left panel). The peak of the similarity distributions at 0.15 indicated that both campaigns achieved high structural diversity. To assess whether the two campaigns explored distinct regions of chemical space, we calculated the pairwise inter-campaign Tanimoto similarity. The peak of this distribution also appeared at 0.15, demonstrating very low similarity between the campaigns.

The same analysis was applied to the accepted molecules from the last generation of each campaign (Fig. 6, right panel). Both campaigns exhibited a shift towards higher Tanimoto similarities and wider distributions, suggesting that the molecules within each campaign became more similar at the final generation. This trend was more pronounced in the constrained campaign (center of the distributions approximately at 0.5), while the free campaign maintained greater diversity (center of the distributions approximately at 0.3). This behavior was expected due to the geometric and chemical restrictions imposed in the constrained campaign. These findings suggest that the constrained campaign explored the local chemical space more intensively than the free campaign but still preserving a degree of diversity. Additionally, the inter-campaign Tanimoto similarity remained low, indicating that the final population of the two campaigns represented different regions of chemical space.

**Fig. 7:**
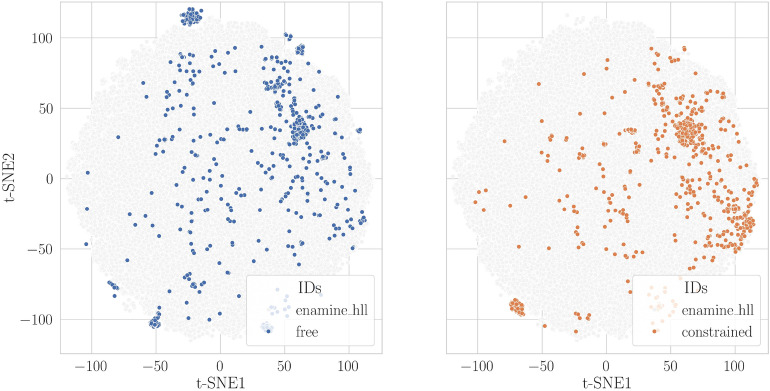
Projection of generated molecules onto the Enamine-Hit Locator Library (HLL-460). The space was constructed based on the Morgan fingerprint of radius 2 and 2048 bits as implemented in RDKit for both the Enamine compounds and all generated molecules, including both accepted and rejected ones, generated during the free and constrained Moldrug campaigns. After calculating the first 50 principal components (PC), which explain 36.7% of the variance, the two main components of the t-distributed stochastic neighbor embedding (t-SNE) were derived from these principal components. For clarity, the free and constrained campaigns are shown in the left and right panels, respectively

To corroborate our previous findings and assess the chemical realism of the designed molecules, we projected all designed molecules (including both accepted and rejected ones) from both campaigns onto the on-demand Enamine-Hit Locator Library (HLL-460), which consists of 460,160 diverse and synthesizable compounds (Fig. 7). This projection also allowed us to estimate which region of chemical space was explored. We used the two main components of the t-distributed Stochastic Neighbor Embedding (t-SNE). In addition, the distribution of *sa_score* and *qed* was calculated for HLL-460 and the molecules of the last generation of each campaign.

Both campaigns effectively explored the chemical space, with the free campaign exhibiting greater diversity. The majority of the designed molecules were located within the region covered by the HLL-460 library (Fig. 7), and the distribution of *sa_score* for the final generation in both campaigns (Fig. S8, right panel) fell within the range of compounds found in HLL-460. These results suggest that the designed molecules are likely synthesizable, supporting the viability of the designs produced by Moldrug.

Notably, uncharted regions of chemical space were also explored, indicating that Moldrug can propose novel molecules. The overlap of *qed* distributions between the molecules from the final generation of both campaigns and the HLL-460 library suggests that the achieved novelty should not come at the expense of drug-likeness. The *qed* distribution for the HLL-460 library skews toward higher values compared to the Moldrug molecules. However, it is important to highlight that the target value for *qed* during optimization was set to 0.75 (Table 1), which explains why the peak of the distributions for both free and constrained campaigns is centered near this value.

The two campaigns explored different regions of this chemical space with denser sampling observed in the region t-SNE1 $\in [50; 100]$. Notably, the constrained simulation, characterized by conserved the heavy atoms of the seed molecule, did not lead to a reduced structural diversity of the designed molecules.

In summary, these results indicate that Moldrug effectively explored diverse regions of chemical space while maintaining computational efficiency, novelty and chemical realism on the proposed molecules. The high structural diversity observed within and between the campaigns and the steady acceptance rates suggests that the algorithm successfully balances exploration and exploitation.

### Evolution of the population

**Fig. 8:**
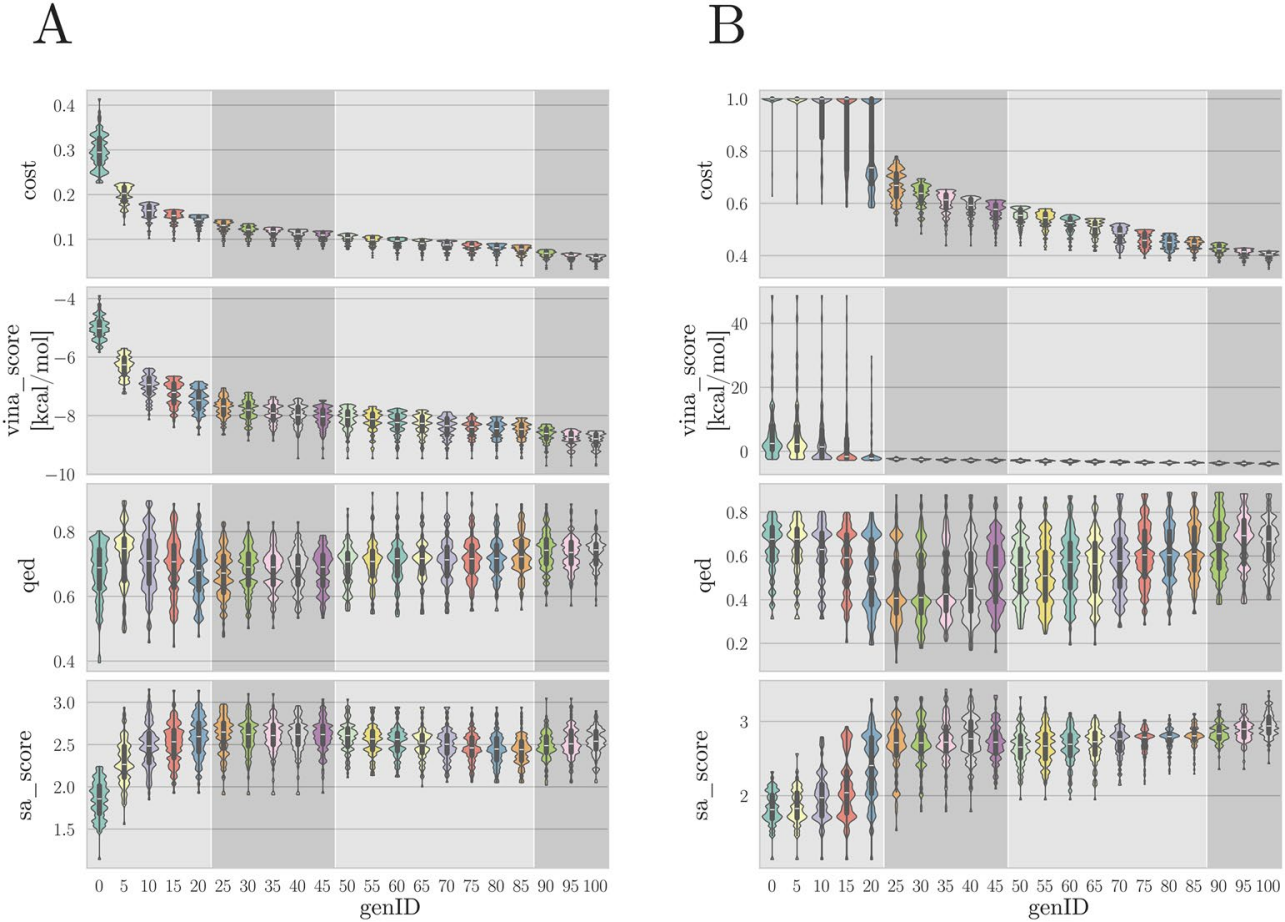
Evolution of the population profile across generations for: (**A**) free and (**B**) constrained campaigns. Shaded regions indicate the four stages followed for the optimization (see the section section Moldrug Parameters). Violin plots were produced with the Seaborn [61] Python library

The evolution of the population across generations was analyzed to assess the effectiveness of Moldrug’s optimization process in guiding molecular design. Specifically, we tracked the progress of the three key properties included in the desirability function: *vina_score*, *qed*, and *sa_score*, along with the consensus score (*cost*), to evaluate how these properties evolved during the simulations. Figure 8 illustrates the progression of these properties across the generations for both the free and constrained campaigns.

#### Designed molecules show high synthesizability

In both free and constrained campaigns, the *sa_score* revealed an initial increase over the first 20 generations, eventually stabilizing and leading to a final population within the range of [2.05; 3.41]; range covered by the HLL-460 library (Fig. S8, right panel). The initial rise of *sa_score* reflected the initial growth phase of the molecules during the first 20 generations, resulting in increasing synthetic complexity. The ability to add or delete heavy atoms in subsequent generations prevented an escalation of synthetic complexity. This property was effectively optimized, considering that the chosen target value for the fitness function was 3 (see Table 1). The best-performing molecules from the free and constrained campaigns exhibited *sa_score* values of 2.38 and 3.10, respectively (see Table 3), indicating that these two molecules are likely synthesizable.

#### Drug-likeness is maintained

The mean *qed* value in the free campaign stabilized around 0.7 with decreasing variability across generations, eventually converging within the range of [0.57; 0.87]. In contrast, the constrained campaign showed greater variability in *qed* values, with the final population spanning a broader range of [0.40; 0.88].

Although the target *qed* value was 0.75, the final populations from both campaigns overlapped well with the *qed* distribution of the HLL-460 library (Fig. S8, left panel), demonstrating that Moldrug is capable of designing drug-like molecules. The best-performing molecules from the free and constrained campaigns achieved *qed* values of 0.78 and 0.73, respectively (Table 3). These outcomes align with the target *qed* value of 0.75, demonstrating that Moldrug effectively optimized for drug-likeness in both campaigns.

#### Challenges in optimizing binding affinity

For the *vina_score*, the optimization shifted the initial population from the range $[-5.84; -3.90] ~\hbox {kcal}\, \hbox {mol}^{-1}$ to a final population within the range $[-9.72; -8.53] ~\hbox {kcal}\, \hbox {mol}^{-1}$ in the free campaign, indicating potent molecules. In contrast, optimizing the *vina_score* proved to be more challenging in the constrained campaign. During the initial 20 generations, some molecules displayed unfavorable binding affinities, resulting in a distribution spanning positive numbers. However, after generation 20, all such molecules were discarded, resulting in a final population with a binding range of $[-4.58, -3.39]~\hbox {kcal}\, \hbox {mol}^{-1}$.

The best-performing molecule in the free campaign achieved a *vina_score* of $-9.23~\hbox {kcal}\, \hbox {mol}^{-1}$, nearing the target value of $-10~\hbox {kcal}\, \hbox {mol}^{-1}$. In contrast, the best-performing molecule in the constrained campaign only reached $-4.28~\hbox {kcal}\, \hbox {mol}^{-1}$. This premature convergence in the constrained campaign may be addressed by modifying the desirability, employing the *local_only* constrained strategy instead of *score_only*, or adjusting the CReM parameters. However, the fine-tuning of these optimizations falls outside the scope of this study.

The best-performing molecule in both campaigns were primarily stabilized by hydrophobic and van der Waals interactions, with polar interactions being relatively underrepresented (Fig. S7 B and D). In the free campaign, the protein-ligand interaction network was more favorable as compared to the constrained campaign, in line with the higher *vina_score*.

Further improvements in polar contacts can be achieved by running additional Moldrug optimization iterations, restricting mutations to the heavy atoms near polar residues in the previously designed molecules. Moreover, Figures S7 A and C indicate that the binding pocket still offers space for molecular growth (see also Fig. S6).

#### The consensus score is optimized

The *cost* value, which represents the weighted desirability of the three properties subtracted from 1 (with 0 being optimal), ranged from [0.033; 0.066] in the free campaign, indicating successful optimization. In contrast, the constrained campaign showed a range of [0.348; 0.417], suggesting only partial optimization. Despite the differences in optimization between the two campaigns, both demonstrated steady evolution toward better molecules. In both cases, all molecules in the last generation outperformed any molecule from its corresponded first generation in terms of *cost* (see Fig. 8, upper panel). This results suggest the effectiveness of Moldrug in optimizing the provided fitness function and the power of the desirability functions for multi-objective optimization.

In this study, the target values for each desirability function were intentionally ambitious, while the upper and lower limits were more permissive. This balance allowed the population to steadily evolve toward better molecules, while still accepting some that were not fully optimized, thus maintaining diversity during the generation process and avoiding premature convergence. Setting overly restrictive limits or ambitious target values from the beginning could risk trapping the optimization process in local minima, particularly if the initial population is not well-suited to such demanding conditions. In practice, a stepwise optimization strategy may be more effective: starting with moderate target values and broader ranges in the desirability functions, allowing the population to explore favorable regions of chemical space, and then gradually refining the parameters to further improve the properties of the molecules.

### Comparison with the literature

The optimization properties were also calculated for the N3 inhibitor. Table 3 illustrates that both best-performing molecules from each campaign outperformed N3 inhibitor in terms of the calculated properties. However, the best-performing molecule from the constrained campaign exhibited a binding affinity that was $2.46 ~\hbox {kcal}\, \hbox {mol}^{-1}$ weaker than N3 after local optimization during docking.

Recently, Luttens et al. [90] screened a diverse library of 235 million virtual compounds against the active site of the M^Pro^ of SARS-CoV-2, followed by fragment-guided optimization of millions of compounds, ultimately identifying 93 candidates, of which eight were confirmed as inhibitor. Here, we calculated the profiles for five of these eight inhibitors for which the authors also provided crystal structures (see Table 3 and Fig. S11). The *qed* and *sa_score* values were comparable to those achieved by the best-performing molecules from both the free and constrained campaigns, although the *qed* values for the inhibitors proposed by [90] were slightly better, indicating superior drug-likeness properties. However, it is important to note that our fixed target value for *qed* in the optimization was set at 0.75.

In terms of docking scores, all 100 molecules from the last population of the free campaign had *vina_scores* lower than $-7.31~\hbox {kcal}\, \hbox {mol}^{-1}$, matching the best docking score achieved (see Table 3) from the five inhibitors proposed by Luttens et al. [90] and tested here. These are encouraging results, as the five compounds were experimentally validated with good outcomes, suggesting that our designed molecules may also prove to be effective inhibitors.

It is also important to highlight that Moldrug did not require the evaluation of hundreds of millions of molecules, as was necessary in Luttens et al. [90]. With only 9,748 designed molecules (both campaigns), we achieved a final population of potential inhibitors comparable to those from virtual screening, based on the discussed properties, and with low similarity to the five inhibitors identified by Luttens et al. [90] that were analyzed in this study (see Fig. S10) This demonstrates that Moldrug is an efficient alternative for exploring chemical space and optimizing molecular properties.

In a recent study, a 3D deep generative model introduced by Li et al. [33] (DeepLigBuilder) designed novel covalent and non-covalent inhibitors targeting the M^Pro^ of SARS-CoV-2. The best-performing covalent compound (compound 4) exhibited *qed* = 0.64, *sa_score* = 3.62, and a Smina score of $-9.2~\hbox {kcal}\, \hbox {mol}^{-1}$. Meanwhile, the leading non-covalent compound (compound 7) displayed *qed* = 0.56, *sa_score* = 2.37, and a Smina score of $-10.7~\hbox {kcal}\, \hbox {mol}^{-1}$.

Among the 100 molecules in the final population of our free campaign, 97 surpassed the best covalent proposed molecule, while 15 exceeded the non-covalent counterpart based on both *qed* and *sa_score*. Assessing docking scores can be challenging; our study utilized AutoDock-Vina, whereas the reference employed Smina (a derivative of AutoDock-Vina) [91]. For AutoDock-Vina, a score below $-9~\hbox {kcal}\, \hbox {mol}^{-1}$ is typically regarded as a threshold for identifying good binders. Based on this criterion, among the 97 molecules outperforming the covalent inhibitor in terms of *qed* and *sa_score*, 25 had *vina_scores* below $-9~\hbox {kcal}\, \hbox {mol}^{-1}$. Furthermore, 5 of the 15 molecules exceeding the non-covalent inhibitor also displayed *vina_scores* lower than $-9~\hbox {kcal}\, \hbox {mol}^{-1}$.

Critically, we do not claim that the designed molecules are superior inhibitors to any of those discussed in this section. As the comparison was based only on the properties considered here (*sa_score*, *qed* and *vina_score*). Experiments instead, are needed to test which of the designed molecules are effective inhibitors in real-life scenarios. It is important to note that more precise fitness functions, designed to better reflect experimental outcomes, can be integrated into the optimization process. This would enhance the accuracy and reliability of predictions for the designed molecules.

**Table 3:** Comparison of the best-performing molecules from free and constrained campaigns with the seed molecule, N3 inhibitor [58], and inhibitors proposed by [90]

**Campaing/PDB ID**	**Molecule structure**	*vina_score* [$\hbox {kcal}\, \hbox {mol}^{-1}$]	*qed*	*sa_score*
Free		$-9.23$	0.78	2.38
Constrained		$-4.28$	0.73	3.10
6LU7 (Seed molecule)		$-1.59$^*b*^	0.57	1.14
6LU7 (N3^*a*^)		$-2.95$^*b*^($-6.74$^*c*^)	0.12	4.29
7AU4		$-6.72$^*c*^	0.86	3.57
7B2J		$-7.31$^*c*^	0.82	2.00
7B2U		$-7.20$^*c*^	0.87	3.07
7B5Z		$-7.24$^*c*^	0.77	2.33
7B77		$-6.42$^*c*^	0.72	2.33

^*a*^ Chemical structure taken from the PDB structure 6LU7. See Figs. S4 and S6 for more details. ^*b*^ Evaluation of PDB structure without optimization by AutoDock-Vina ^*c*^ Evaluation of PDB structure with local optimization by AutoDock-Vina

### Ranking moldrug hypothesis by molecular dynamic simulations

AutoDock-Vina excels in efficient global conformational exploration at relatively low computational cost but is not without limitations [46]. The reduced computational expense is accompanied by lower accuracy in binding free energy estimations (See Fig. S13). Moldrug uses AutoDock-Vina as its default docking scheme, based on the hypothesis that the AutoDock-Vina scoring function will, at the very least, facilitate the enrichment of the population with good binders.

#### Prioritization with MM/GBSA calculations

To evaluate our hypothesis, the final populations from four key stages (generations 20, 45, 85, and 100) of each campaign were subjected to MM/GBSA calculations. The top 100 globally ranked molecules on each campaign were then selected for alchemical binding free energy calculations.

Figure S12 illustrates the distribution of MM/GBSA scores. The mean MM/GBSA scores for both campaigns improved in the later generations, reflecting optimization progress. However, all generations displayed a broad distribution of MM/GBSA scores, highlighting significant variability within the populations.

#### Ranking with Alchemical binding free energy calculations

From the top 100 molecules ranked by MM/GBSA, duplicates and molecules unsuitable for relative binding free energy (RBFE) calculations were excluded. As a result, 82 and 88 molecules from the free and constrained campaigns, respectively, were selected for RBFE calculations.

RBFEs were converted to absolute binding free energies (ABFEs) by performing ABFE calculations on a representative molecule from each campaign (Tables S1 and S2). Additionally, the ABFE of the N3 inhibitor from the crystal structure proposed by Jin et al. was calculated to be $-6.22~\hbox {kcal}\, \hbox {mol}^{-1}$, facilitating a direct comparison between the designed molecules and the crystal structure molecule based on alchemical binding free energy calculations.

**Fig. 9:**
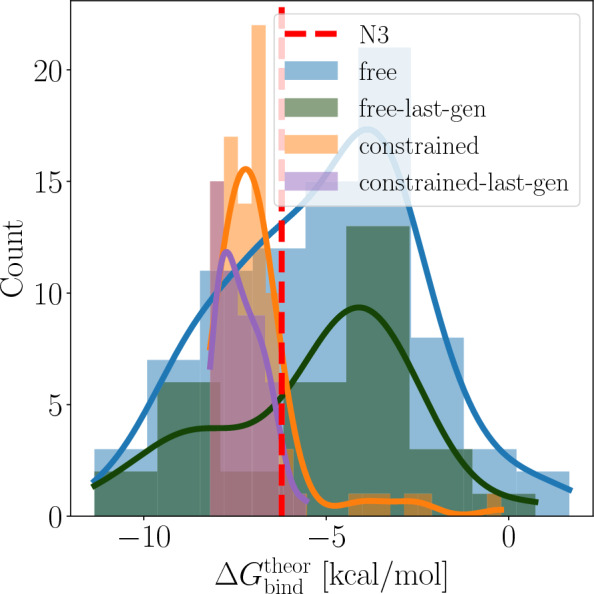
Distribution of calculated absolute binding free energies ($\Delta G_{\textrm{bind}}^{\textrm{theor}}$) for the top-ranked molecules identified in the free and constrained campaigns using MM/GBSA calculations. After excluding duplicates and molecules unsuitable for alchemical binding free energy calculations, 82 and 88 molecules from the respective campaigns were selected for alchemical binding free energy calculations. Among these, 29 and 79 molecules from the free and constrained campaigns, respectively, demonstrated superior $\Delta G_{\textrm{bind}}^{\textrm{theor}}$ compared to the N3 inhibitor from the crystal structure proposed by [88]. Notably, 10 and 51 of these molecules originated from the final generation of the free and constrained campaigns

Figure 9 presents the distribution of ABFE values. Of the designed molecules, 29 and 79 from the free and constrained campaigns, respectively, achieved better binding free energies than the N3 inhibitor (Tables S1 and S2). Of these, 10 and 51 were found in the final generation of the free and constrained campaigns (Figures S14 and S15). In a recent study by Li et al. [92], 25 putative inhibitors of the M^Pro^ of SARS-CoV-2 were identified using alchemical binding free energy calculation, 15 of which were experimentally confirmed. Among these, Dipyridamole [93] exhibited the highest experimental binding free energy at $-10.1~\hbox {kcal}\, \hbox {mol}^{-1}$. Notably, molecules f-4916, f-4329, and f-799 (the first two appearing in the final generation of the free campaign) were predicted to have comparable binding free energies (Table S1). These results underscore Moldrug’s ability to enrich the population with potent binders using AutoDock-Vina while simultaneously optimizing *sa_score* and *qed* profiles.

Interestingly, while the free campaign produced the most potent molecules and a wider distribution of binding free energies, the constrained campaig exhibited a distribution skewed towards better binders. This difference could be attributed to the the greater diversity retained in the final population of the free campaign (Fig. 6, right panel), indicating that the optimization, while converged in terms of the consensus score (*cost*), has not yet fully converged on a specific chemical structure. By contrast, the constrained campaign, due to geometric and chemical restrictions, explored the local chemical space more intensively, allowing the identification of stronger binders within that region. This is supported by the shift toward higher similarity values observed in the constrained campaign (Fig. 6, right panel).

Despite these promising results, Fig. S13 reveal a weak correlation between AutoDock-Vina scores and ABFE values, emphasizing the need for more sophisticated methods like alchemical binding free energy for accurate potency ranking. The constrained campaign, in particular, showed underestimated *vina_scores* (Fig. 9-b), possibly due to the use of the *score_only* scheme, which may not be optimal for precise docking evaluations, and the high similarity among molecules (Fig. 6, right panel), which likely limits AutoDock-Vina to discriminate between the molecules. The *local_only* scheme could yield more accurate results. However, these limitations were effectively addressed during molecular dynamics simulations, which successfully distinguished between molecules.

While increasing ligand size is a straightforward approach to enhance potency, it often results in molecules unsuitable for pharmaceutical applications. In our simulations, the number of atoms stabilized after the first 20 generations (Fig. S9, where ligand growth was favored. This stabilization reflects the influence of *sa_score* and *qed*, which indirectly regulate against unnecessary growth, ensuring molecules are optimized to exploit key interactions rather than merely increasing in size.

These results highlight the potential of the Moldrug approach when combined with free energy calculations. This integration improves the ranking of designed molecules, as demonstrated in previous studies [94–97], enhancing the reliability of selecting putative drugs for experimental testing while reducing the burden of unnecessary evaluations.

## Conclusions

We introduced Moldrug, a software and algorithm designed to identify putative binders by exploring the chemical space. Moldrug explores the chemical space with a genetic algorithm, where molecules are generated by chemically guided mutations suggested by the CReM library and ranked by an adaptable fitness function. Molecules were, in this study, designed with the aim of finding a reasonable consensus between (i) binding affinity as quantified by a AutoDock-Vina docking score, (ii) drug-likeness quantified by qed, and (iii) synthetic accessibility quantified by the sa_score. Moldrug may optimize the provided fitness function starting from a hit compound with a known binding pose, optionally constraining the binding pose of the hit and/or a substructure of the molecule. Without constraints, Moldrug has the maximum freedom for designing molecules. If no hit compound is available, the optimization can start from any small molecule, such a methane. Designed molecules, along with their binding poses and property profiles, can be interactively explored using Moldrug-Dashboard.

Moldrug is a free software published under the Apache 2.0 license. Moldrug is developed openly on GitHub at https://github.com/ale94mleon/moldrug. Documentation and tutorials are available at https://moldrug.rtfd.io.

We demonstrated the capabilities of Moldrug by designing potential inhibitors for the main protease (M^Pro^) of SARS-CoV-2. The designed molecules exhibited high chemical diversity, as indicated by low Tanimoto similarity scores and by comparison with the Enamine HLL-460 chemical space.

Binding free energy calculations using MM/GBSA or alchemical transformations, as used here, were essential for accurate ranking of the designed molecules. We identified 108 molecules with predicted affinities equal to or better than the N3 inhibitor, with a predicted affinity of $-6.22~\hbox {kcal}\, \hbox {mol}^{-1}$. These molecules achieved binding free energies as low as $-10~\hbox {kcal}\, \hbox {mol}^{-1}$.

Moldrug has been designed to be highly adaptable. New fitness functions may be implemented and shared with the community by adding them to the Contrib directory of the Moldrug repository, thereby enabling the evaluation of molecules with free or commercial software. For instance, a structure-activity relation (SAR) score for a specific target may be added to bias the chemical space sampling. Binding pose prediction or scoring may be carried out with AI-based tools such as AlphaFold 3 [98] instead of AutoDock-Vina as used here. Free energy calculation techniques such as MM/GBSA may be used instead of the docking score to achieve more accurate affinity prediction at the price of a higher computational cost.

The modularity, flexibility, and open license of Moldrug are anticipated to be advantageous for various drug design projects, spanning from hit identification to lead generation and optimization. However, by modifying the database of interchangeable fragments to target specific regions of chemical space, combined with the native integration of user-customized fitness functions, Moldrug may provide a versatile framework for molecular and material design.

## Supplementary Information

Supplementary Material 1

## Data Availability

$\bullet$ **Project name**: moldrug $\bullet$ **Project home page**: https://github.com/ale94mleon/moldrug $\bullet$ **Operating system**: Platform independent $\bullet$ **Programming language**: Python $\bullet$ **Other requirements**: rdkit$>=$2022.3.5, crem, tqdm, numpy, pandas, pyyaml, dill, meeko, six, scipy. All listed in the main project and automatically installed. $\bullet$ **License**: Apache 2.0 license

## Abbreviations

qed: Quantitative Estimation of Drug-likeness
sa_score: Synthetic Accessibility Score
AI: Artificial Intelligence
ML: Machine Learning
CPU: Central processing unit
GPU: Graphics processing unit
SMILES: Simplified Molecular Input Line Entry System
CReM: Chemically Realistic Enumeration of Molecules
GA: Genetic Algorithm
CLI: Command Line Interface
$\textrm{pH} = -\log [\mathrm {H^+}]$: where $[\mathrm {H^+}]$ is the molar concentration of hydrogen ions in the solution
KDE: Kernel Density Estimation
$$\textrm{M}^{\textrm{Pro}}$$: Main Protease
HLL-460: Enamine-Hit Locator Library (460,160 compounds)
PCA: Principal Component Analysis
t-SNE: t-distributed Stochastic Neighbor Embedding
AM1-BCC: Austin Model 1 - Bond Charge Corrections
HMR: Hydrogen Mass Repartitioning
MM/GBSA: Molecular Mechanics Generalized-Born Surface Area
RBFE: Relative Binding Free Energy
ABFE: Absolute Binding Free Energy
HDBSCAN: Hierarchical Density-Based Spatial Clustering of Applications with Noise
