# Supplementary material for: Moldrug algorithm for an automated ligand binding site exploration by 3D aware molecular enumerations
Source: J Cheminform. 2025 May 26;17:85. doi: 10.1186/s13321-025-01022-3 (PMC12107812; doi:10.1186/s13321-025-01022-3)
Supplement: Supplementary file 1 — Supplementary Material 1 [file 13321_2025_1022_MOESM1_ESM.pdf]

Moldrug algorithm for an automated ligand binding site exploration by 3D aware molecular enumerations

Alejandro Martínez León,[*,†] Benjamin Ries,[‡,¶] Jochen S. Hub,[*,†] and Aniket Magarkar[*,‡]

†*Theoretical Physics and Center for Biophysics, Universität des Saarlandes, PharmaScienceHub (PSH), 66123 Saarbrücken, Germany*

‡*Boehringer Ingelheim Pharma GmbH & Co KG, Medicinal Chemistry, Birkendorfer Str. 65, 88397 Biberach an der Riss, Germany*

¶*Open Free Energy, Open Molecular Software Foundation, Davis, CA, 95616, United States*

E-mail: alejandro.martinezleon@uni-saarland.de; jochen.hub@uni-saarland.de; aniket.magarkar@boehringer-ingelheim.com

Abstract

We present Moldrug, a computational tool for accelerating the hit-to-lead phase in structure-based drug design. Moldrug explores the chemical space using structural modifications suggested by the CReM library and by optimizing an adaptable fitness function with a genetic algorithm. To illustrate Moldrug, we designed new potential inhibitors targeting the main protease (M^{Pro}) of SARS-CoV-2 by optimizing a consensus fitness function that balances binding affinity, drug-likeness, and synthetic accessibility. The designed molecules exhibited high chemical diversity. The designed molecules were ranked using MM/GBSA and alchemical binding free energy calculations, revealing predicted affinities as low as $-10\ \mathrm{kcal\,mol^{-1}}$. Moldrug is distributed as a Python

package under the Apache 2.0 license. It offers a pre-configured multi-parameter fitness functions for ligand optimization, while being highly adaptable for integrating functionalities from external software. Documentation and tutorials are available at https://moldrug.rtfd.io. Visual inspection of designed molecules is provided by a user-friendly web application Moldrug-Dashboard.

Scientific contribution. Moldrug is a modular and flexible open-source framework for efficient exploration of the chemical space without need of prior training, primarily focused on the design of novel inhibitors. We demonstrated Moldrug by designing new potential inhibitors for the M^{Pro} of SARS-CoV-2 with high predicted affinity according to alchemical free energy calculations. Moldrug follows good coding practice and is accompanied by detailed documentation, making Moldrug an accessible and adaptable resource for cheminformatics research.

Supporting Information

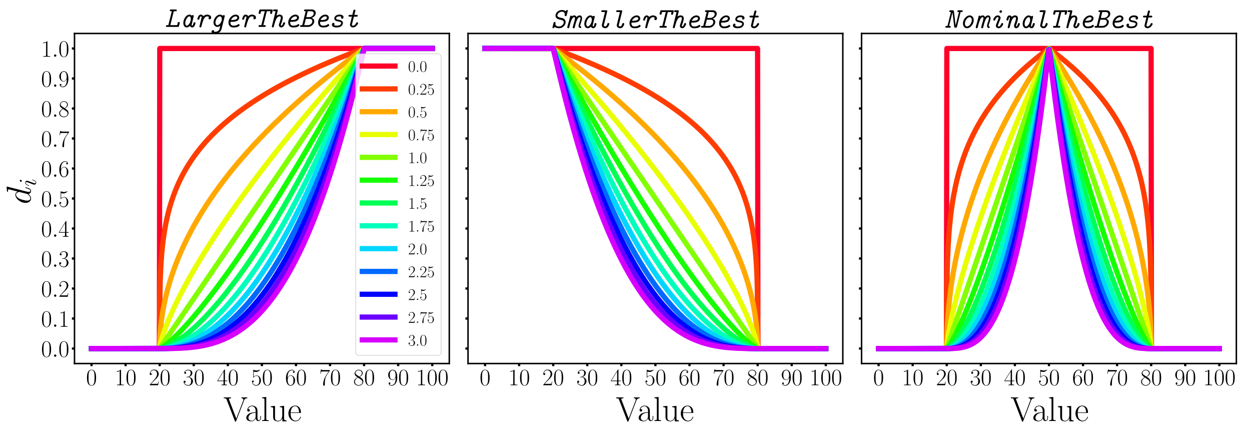

Figure S1: Dependency of each desirability with the exponent r in the interval $[0; 100]$. `LargerTheBest`: with $LowerLimit = 20$ and $Target = 80$. `SmallerTheBest`: $Target = 20$ and $UpperLimit = 80$. `NominalTheBest`: $LowerLimit = 20$, $Target = 50$ and $UpperLimit = 80$.

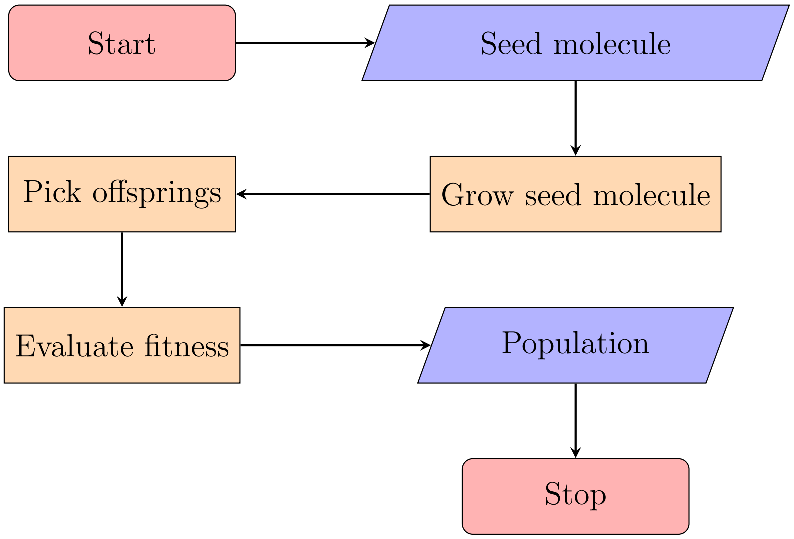

Figure S2: Flowchart of `Local` class

```python
from moldrug import utils, fitness
from rdkit import Chem
# Initialize the class
out = utils.Local(
    seed_mol=Chem.MolFromSmiles( 'CC(O)CNC(CC)C'),
    costfunc=fitness.Cost,
    costfunc_kwargs={
        'vina_executable':
        ↪  <path/to/vina/executable/file>,
        'receptor_pdbqt_path': <path/to/receptor.pdbqt>,
        'boxcenter' : [<center_x>, <center_y>,
        ↪  <center_z>],
        'boxsize': [<size_x>, <size_y>, <size_z>],
        'ncores': 4},
    crem_db_path = <path/to/cream_fragment.db>,
    grow_crem_kwargs={
        'radius': 3,
        'min_atoms': 0,
        'max_atoms': 8,
        'ncores': 12})
# Start simulation. Pick a maximum of 100 of the generated
↪  structures
out(njobs=3, pick=100)
# Save on disk
out.pickle('local', compress=True)
```

```yaml
local:
  type: Local
  njobs: 3
  pick: 100
  seed_mol:  "CC(O)CNC(CC)C"
  costfunc: Cost
  costfunc_kwargs:
    vina_executable:
    ↪  <path/to/vina/executable/file>
    receptor_pdbqt_path:
    ↪  <path/to/receptor.pdbqt>
    boxcenter:
      - <center_x>
      - <center_y>
      - <center_z>
    boxsize:
      - <size_x>
      - <size_y>
      - <size_z>
    ncores: 4
  crem_db_path:
  ↪  <path/to/cream_fragment.db>
  grow_crem_kwargs:
    radius: 3
    min_atoms: 0
    max_atoms: 8
    ncores: 12
  deffnm: local
```

Figure S3: Demonstrating the utilization of the *Local* class in Moldrug. The example includes initialization, calling of the class and saving to disk. **Left**: Python code snippet, **Right**: configuration file for the command line interface. Only *seed_mol*, *costfunc*, *costfunc_kwargs* and *crem_db_path* are mandatory parameters during the initialization of the class.

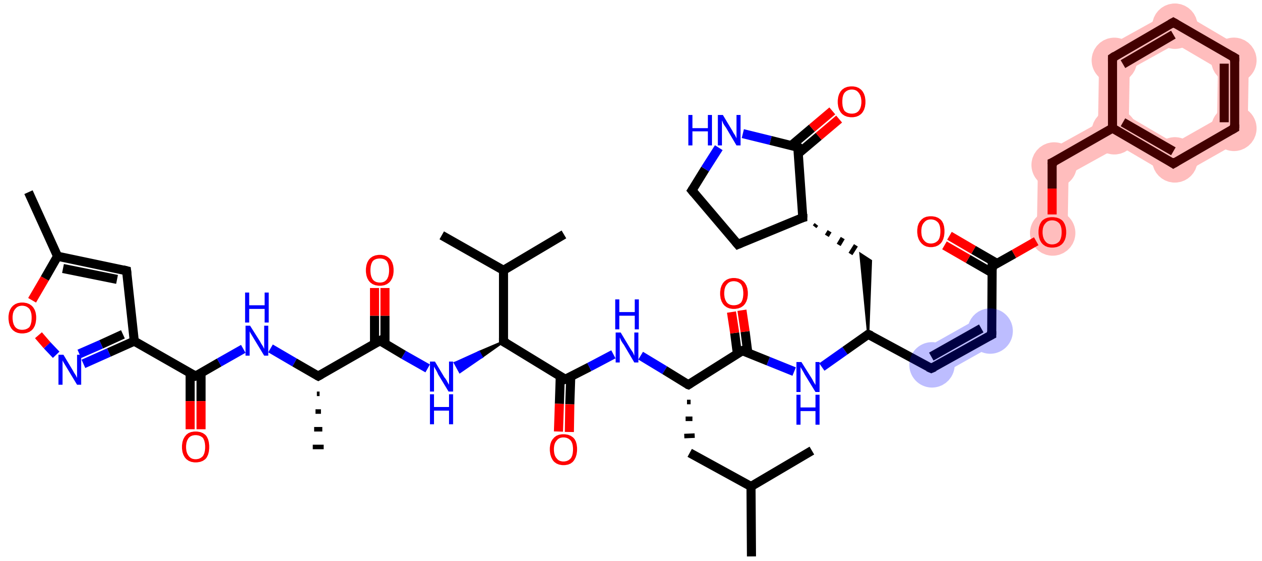

Figure S4: The chemical structure is taken from the PDB 6LU7.[1] The showed stereochemistry is inferred by RDKit[2] from the conformation presented in the crystal structure. The double bond close to the phenylacetyl fragment (blue highlight) was manually added following its description in the main publication.[1] Highlighted in red is the fragment of the inhibitor used as the seed molecule for the Moldrug simulations.

Table S1: Absolute binding free energy (ABFE) results for the final 82 molecules submitted to free energy perturbation in the free campaign. RBFE values were converted to ABFE by estimating the ABFE of the representative molecule, f-3964 (highlighted in bold in the table). See the Methods section for further details.

idx	ΔG_{bind} [kcal mol^{-1}]	idx	ΔG_{bind} [kcal mol^{-1}]
f-4916	-11.35 ± 0.9	f-4794	-4.47 ± 0.97
f-4329	-10.25 ± 0.9	f-3495	-4.4 ± 0.82
f-799	-10.16 ± 0.65	f-4805	-4.39 ± 0.85
f-2713	-9.27 ± 0.77	f-4088	-4.34 ± 0.87
f-4132	-9.26 ± 0.79	f-4541	-4.3 ± 0.76
f-4288	-9.24 ± 0.79	f-932	-4.21 ± 0.81
f-4814	-8.95 ± 0.81	f-3503	-4.17 ± 0.73
f-4922	-8.89 ± 0.71	f-4890	-4.08 ± 0.83
f-4477	-8.67 ± 1.02	f-3237	-3.97 ± 0.72

idx (cont.)	ΔG_{bind} [kcal mol^{-1}] (cont.)	idx (cont.)	ΔG_{bind} [kcal mol^{-1}] (cont.)
f-1136	-8.53 ± 0.64	f-4299	-3.94 ± 0.65
f-3969	-8.42 ± 0.75	f-816	-3.83 ± 0.77
f-4905	-8.35 ± 0.97	f-3793	-3.82 ± 0.97
f-3769	-8.31 ± 1.01	f-4832	-3.65 ± 0.82
f-1546	-8.05 ± 0.63	f-4720	-3.65 ± 0.71
f-4736	-8.04 ± 0.77	f-1239	-3.6 ± 0.81
f-3279	-7.47 ± 0.73	f-3091	-3.57 ± 0.83
f-2149	-7.43 ± 0.77	f-862	-3.55 ± 1.02
f-548	-7.36 ± 0.69	f-1075	-3.55 ± 0.65
f-4941	-7.31 ± 0.75	**f-3964**	-3.52 ± 0.69
f-702	-7.1 ± 0.65	f-4939	-3.51 ± 0.73
f-2064	-7.07 ± 1.01	f-4701	-3.5 ± 0.77
f-3563	-6.86 ± 0.81	f-566	-3.48 ± 0.86
f-979	-6.75 ± 0.87	f-3946	-3.46 ± 0.74
f-2823	-6.68 ± 0.98	f-457	-3.24 ± 0.73
f-4146	-6.51 ± 0.77	f-1855	-3.1 ± 0.88
f-700	-6.4 ± 0.83	f-4741	-3.03 ± 0.73
f-4913	-6.37 ± 0.82	f-4014	-2.94 ± 0.74
f-2026	-6.36 ± 0.85	f-871	-2.72 ± 0.83
f-3673	-6.31 ± 0.75	f-4540	-2.58 ± 0.85
f-2063	-6.14 ± 0.79	f-3214	-2.57 ± 0.76
f-2112	-5.97 ± 0.71	f-3200	-2.44 ± 0.73
f-4551	-5.76 ± 0.79	f-4774	-2.42 ± 1.15
f-2004	-5.67 ± 0.7	f-4914	-2.32 ± 0.56
f-4617	-5.55 ± 0.77	f-1743	-2.25 ± 0.77
f-866	-5.52 ± 1.03	f-3716	-1.56 ± 0.96

idx (cont.)	ΔG_{bind} [kcal mol^{-1}] (cont.)	idx (cont.)	ΔG_{bind} [kcal mol^{-1}] (cont.)
f-1813	−5.51 ± 0.87	f-2259	−1.41 ± 0.85
f-2200	−5.36 ± 0.8	f-1048	−1.11 ± 0.83
f-2548	−5.33 ± 0.69	f-631	−0.83 ± 0.84
f-4695	−5.26 ± 1.19	f-1767	0.18 ± 0.84
f-4264	−5.15 ± 0.81	f-2827	0.73 ± 0.92
f-4046	−4.97 ± 0.78	f-2111	1.66 ± 1.0

Table S2: Absolute binding free energy (ABFE) results for the final 88 molecules submitted to free energy perturbation in the constrained campaign. RBFE values were converted to ABFE by estimating the ABFE of the representative molecule, c-4399 (highlighted in bold in the table). See the Methods section for further details.

idx	ΔG_{bind} [kcal mol^{-1}]	idx	ΔG_{bind} [kcal mol^{-1}]
c-4705	−8.18 ± 0.44	c-4498	−7.16 ± 0.21
c-4568	−8.16 ± 0.35	c-4373	−7.06 ± 0.14
c-4279	−8.15 ± 0.19	c-4127	−7.04 ± 0.17
c-4623	−8.05 ± 0.16	c-3710	−7.03 ± 0.49
c-4685	−8.01 ± 0.25	c-3378	−6.98 ± 0.29
c-4630	−7.99 ± 0.73	c-3683	−6.96 ± 0.58
c-4612	−7.97 ± 0.9	c-3889	−6.94 ± 0.3
c-4298	−7.94 ± 0.24	c-3634	−6.92 ± 0.29
c-4733	−7.94 ± 0.87	c-3804	−6.89 ± 0.33
c-4552	−7.93 ± 0.41	c-4593	−6.88 ± 0.29
c-4739	−7.88 ± 0.22	c-2652	−6.87 ± 0.32
c-4693	−7.85 ± 0.18	c-3762	−6.87 ± 0.55
c-4348	−7.83 ± 0.25	c-4576	−6.86 ± 0.19
c-4639	−7.82 ± 0.67	c-3817	−6.83 ± 0.17
c-4750	−7.81 ± 0.44	c-3779	−6.82 ± 0.22

idx (cont.)	ΔG_{bind} [kcal mol^{-1}] (cont.)	idx (cont.)	ΔG_{bind} [kcal mol^{-1}] (cont.)
c-4358	-7.8 ± 0.47	c-3603	-6.81 ± 0.69
c-4731	-7.74 ± 0.65	c-4582	-6.81 ± 0.38
c-4783	-7.71 ± 0.2	c-4764	-6.8 ± 0.4
c-4254	-7.69 ± 0.45	c-3627	-6.79 ± 0.19
c-4534	-7.67 ± 0.57	c-3425	-6.78 ± 0.23
c-3559	-7.65 ± 0.56	c-4038	-6.77 ± 0.26
c-4509	-7.62 ± 0.45	c-3995	-6.73 ± 0.41
c-4549	-7.58 ± 0.29	c-3464	-6.71 ± 0.26
c-4513	-7.5 ± 0.38	c-3670	-6.68 ± 0.11
c-3915	-7.49 ± 0.3	c-4562	-6.65 ± 0.15
c-4497	-7.49 ± 0.32	c-3668	-6.64 ± 0.54
c-3685	-7.48 ± 0.39	c-4414	-6.62 ± 0.38
c-4432	-7.47 ± 0.11	c-4422	-6.49 ± 0.17
c-3997	-7.47 ± 0.35	c-4133	-6.45 ± 0.35
c-2623	-7.45 ± 0.17	c-3563	-6.44 ± 0.1
**c-4399**	-7.45 ± 0.1	c-4064	-6.43 ± 0.27
c-3721	-7.45 ± 0.37	c-3309	-6.33 ± 0.26
c-3335	-7.38 ± 0.22	c-3173	-6.33 ± 0.49
c-4710	-7.37 ± 0.22	c-4777	-6.3 ± 0.81
c-4761	-7.34 ± 0.33	c-4016	-6.27 ± 0.59
c-3346	-7.28 ± 0.25	c-4717	-6.03 ± 0.85
c-2858	-7.25 ± 0.32	c-3318	-5.98 ± 0.49
c-2915	-7.25 ± 0.33	c-4768	-5.55 ± 0.28
c-4659	-7.24 ± 0.3	c-980	-4.37 ± 0.89
c-4789	-7.24 ± 0.45	c-1903	-3.93 ± 1.4
c-4218	-7.22 ± 0.53	c-1595	-3.38 ± 2.06

idx (cont.)	ΔG_{bind} [kcal mol^{-1}] (cont.)	idx (cont.)	ΔG_{bind} [kcal mol^{-1}] (cont.)
c-4323	-7.22 ± 0.42	c-1025	-2.66 ± 0.66
c-4079	-7.21 ± 0.14	c-2006	-2.37 ± 1.17
c-3368	-7.19 ± 0.23	c-737	-0.21 ± 1.27

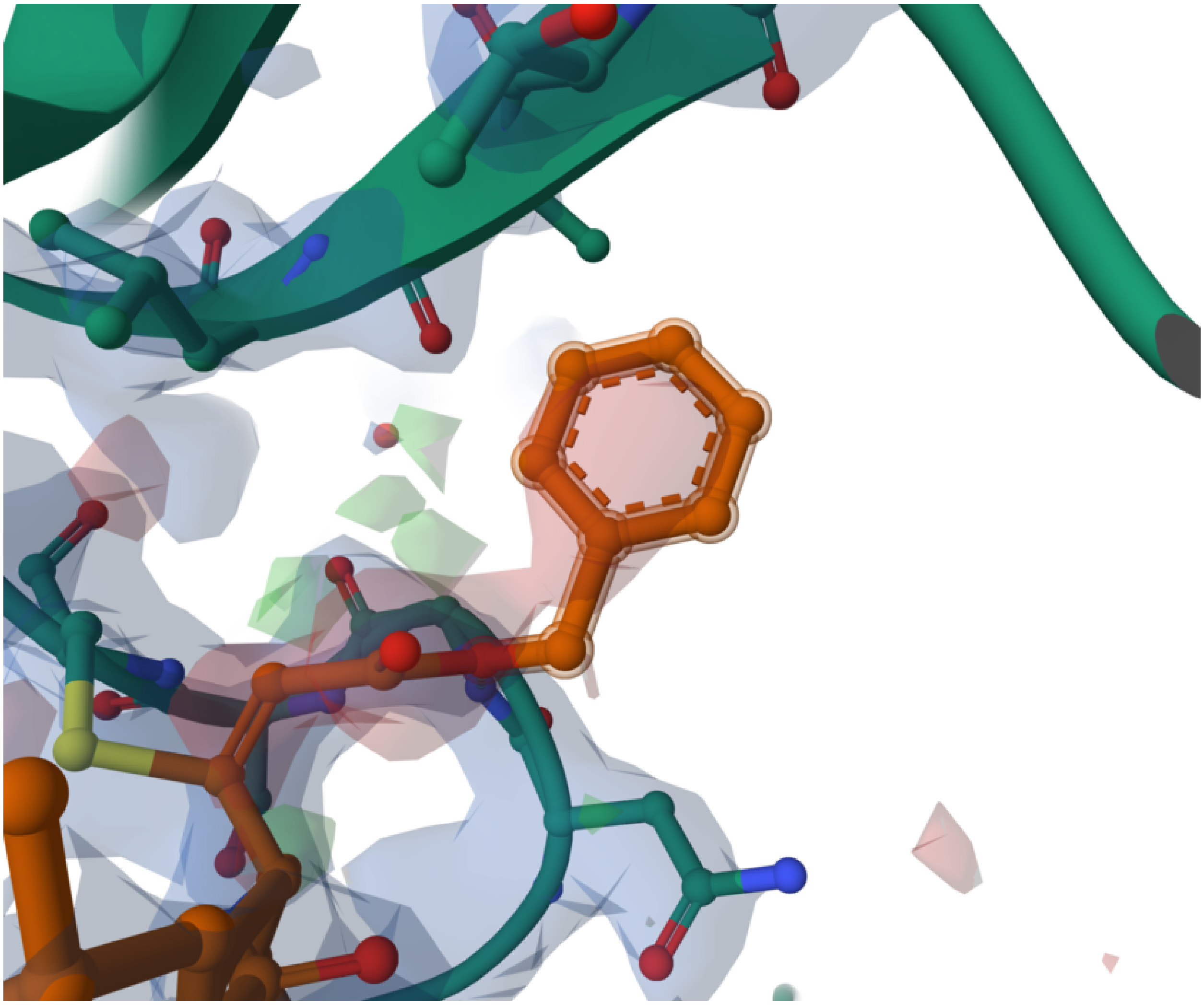

Figure S5: Density maps illustrating the molecular interactions of the M^{Pro} from SARS-CoV-2, represented by green cartoon and sticks, in complex with the N3 inhibitor (depicted in orange sticks). The 2Fo-Fc electron density map ($\sigma = 1.5$) is shown in blue, while the positive Fo-Fc electron density map ($\sigma = 3$) is presented in green, and the negative Fo-Fc electron density map ($\sigma = -3$) is highlighted in red. Notably, the experimental density does not adequately describe the 2-phenylethanol fragment of the N3 inhibitor. This figure was generated using the PDB server: https://www.rcsb.org/3d-view/6LU7.

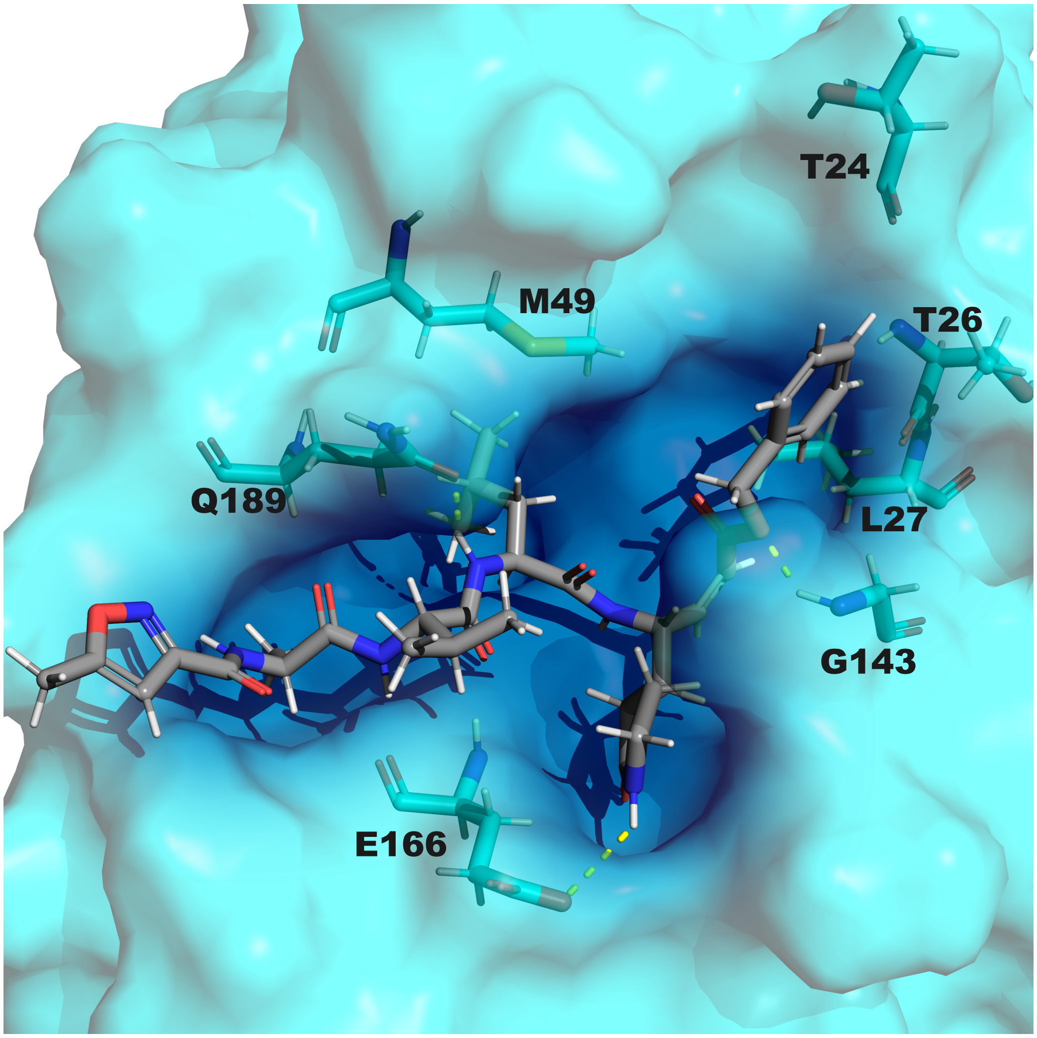

Figure S6: Protein-ligand complex formed by N3 inhibitor with the M^{Pro} of SARS-CoV-2. The binding pocket is highlighted in dark blue. Relevant polar interactions are highlighted in dotted yellow lines. The images were generated with PyMOL.[3]

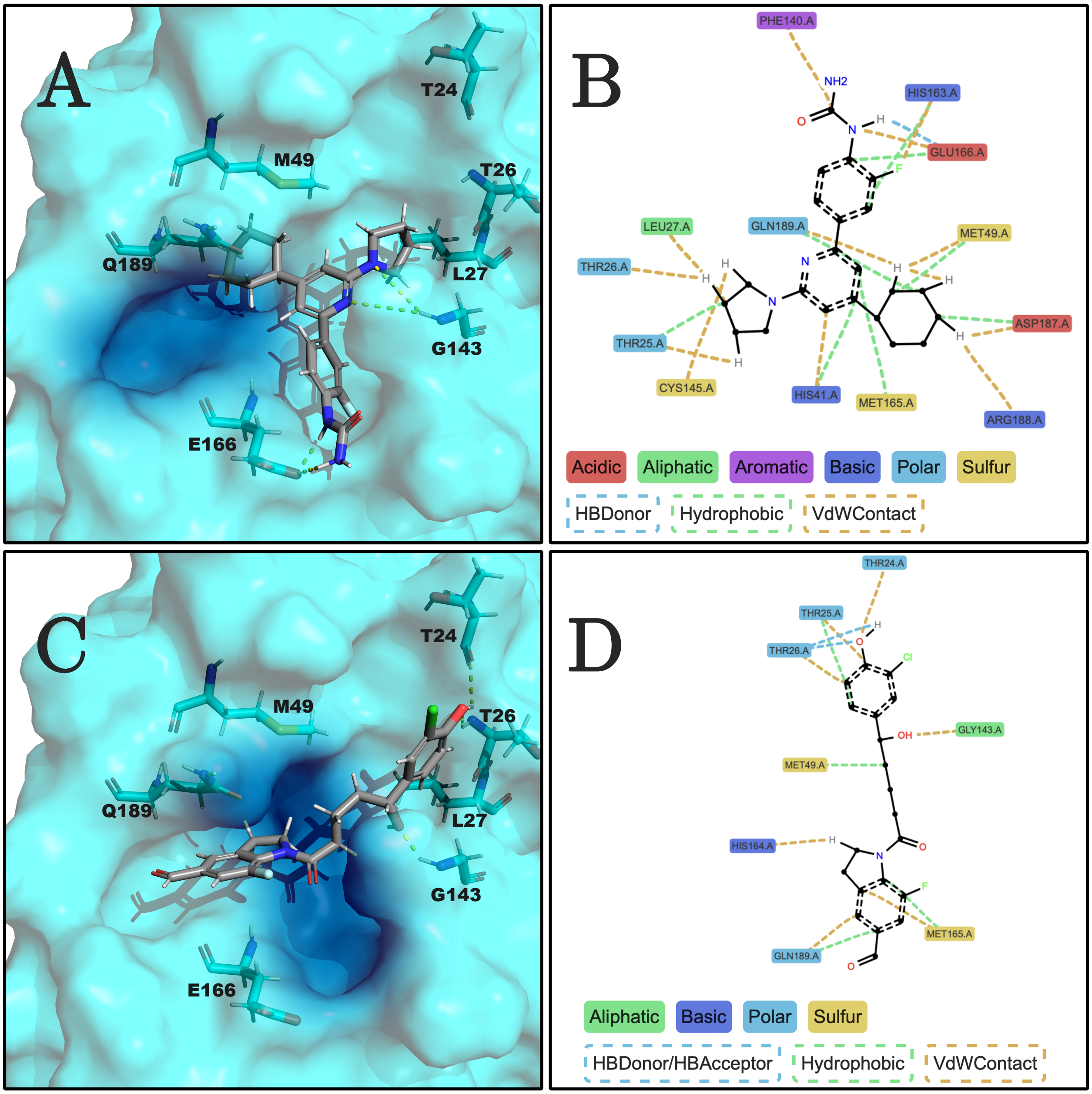

Figure S7: Protein-ligand complex formed by the best molecule propoused by Moldrug on each campaign with the M^{Pro} of SARS-CoV-2. Panels **A** and **C** depict the predicted three-dimensional conformation for the free and constrained campaigns, respectively. In dark blue, the regions of the binding pocket in which the molecule might still grow. Panels **A** and **C** shows relevant polar interactions as dotted yellow lines. The images were generated with PyMOL.[3] Panels **B** and **D** display the protein-ligand interaction network created by ProLIF[4] for the free and constrained campaigns, respectively.

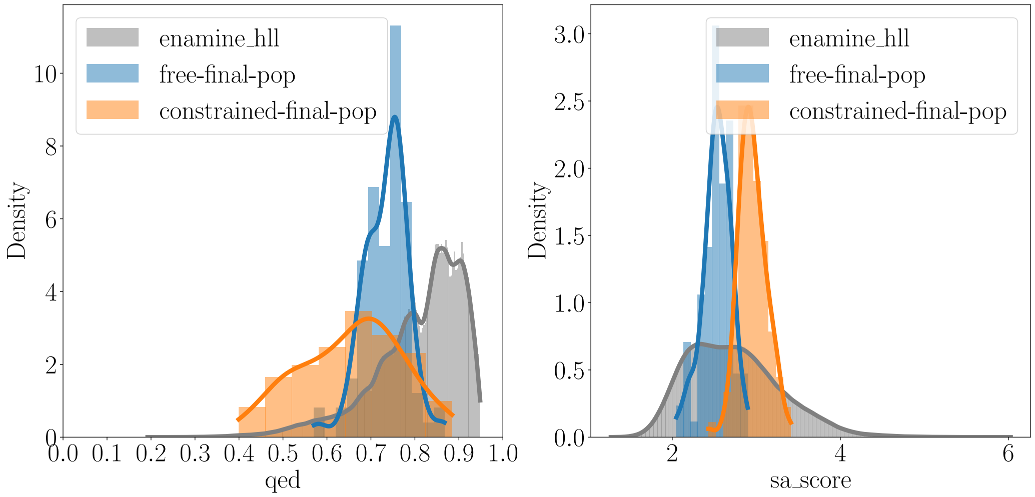

Figure S8: Quantitative Estimation of Drug-likeness (*qed*, left panel) and Synthetic Accessibility Score (*sa_score*, right panel) distributions for the molecules of the last generation of each campaign and the compounds in the Enamine-Hit Locator Library (HLL-460). For clarity, lines represent smoothed histograms, computed using kernel density estimation (KDE). Seaborn[5] Python library was used to construct the histograms. Number of bins were automatic selected by Seaborn.

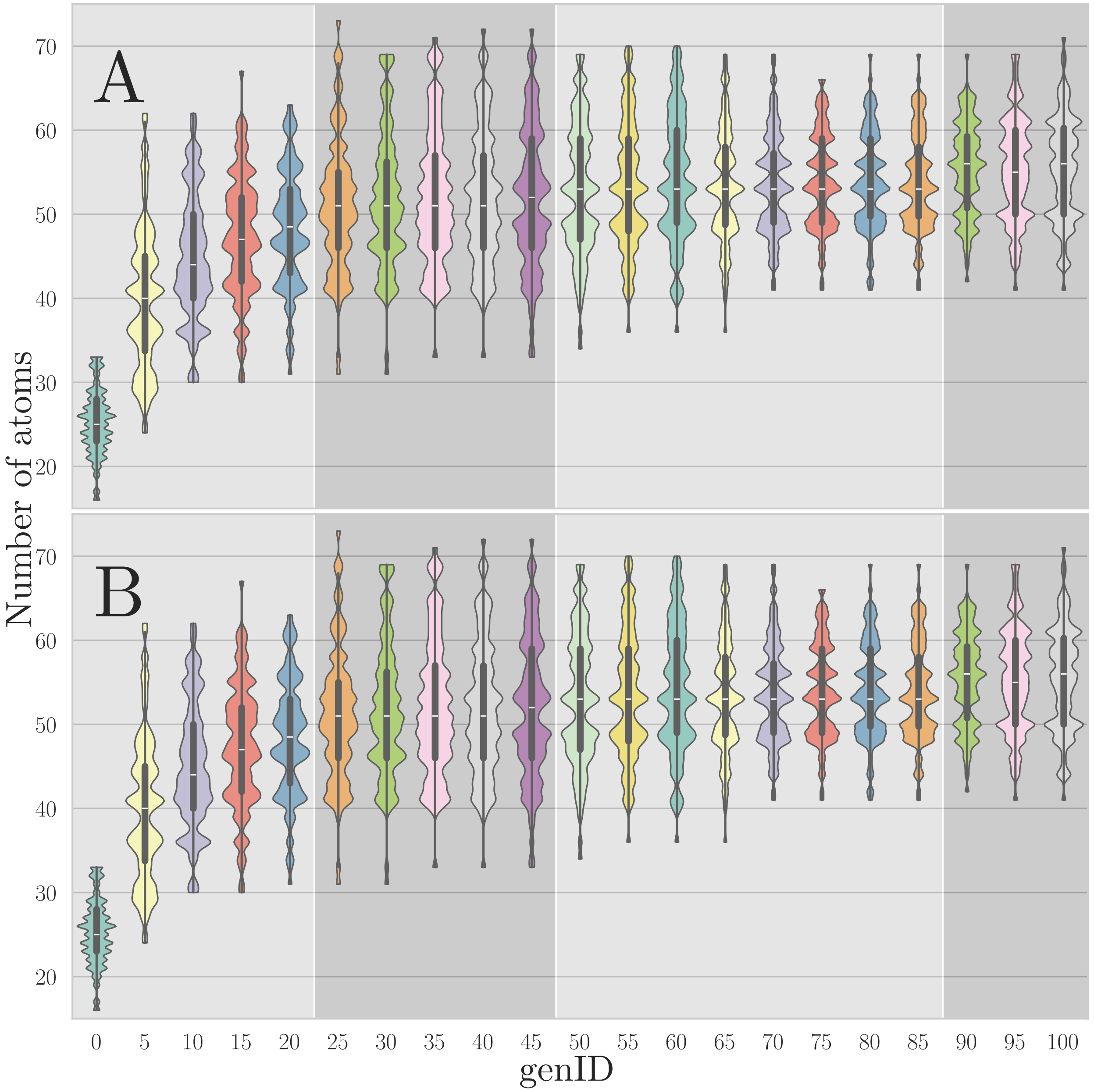

Figure S9: Evolution of the number of atoms (heavy and hydrogen atoms) across generations for: **A** free and **B** constrained campaigns. Shaded regions represent the four stages of optimization (see the section Moldrug Parameters for more details). During the first 45 generations, the distribution shifts towards a higher number of atoms, particularly in the constrained campaign. This shift is primarily due to the CReM parameters used in the first two stages, which favored ligand growth. Over the subsequent generations, the number of heavy atoms stabilized across the populations. Violin plots were generated using the Seaborn Python library.[5]

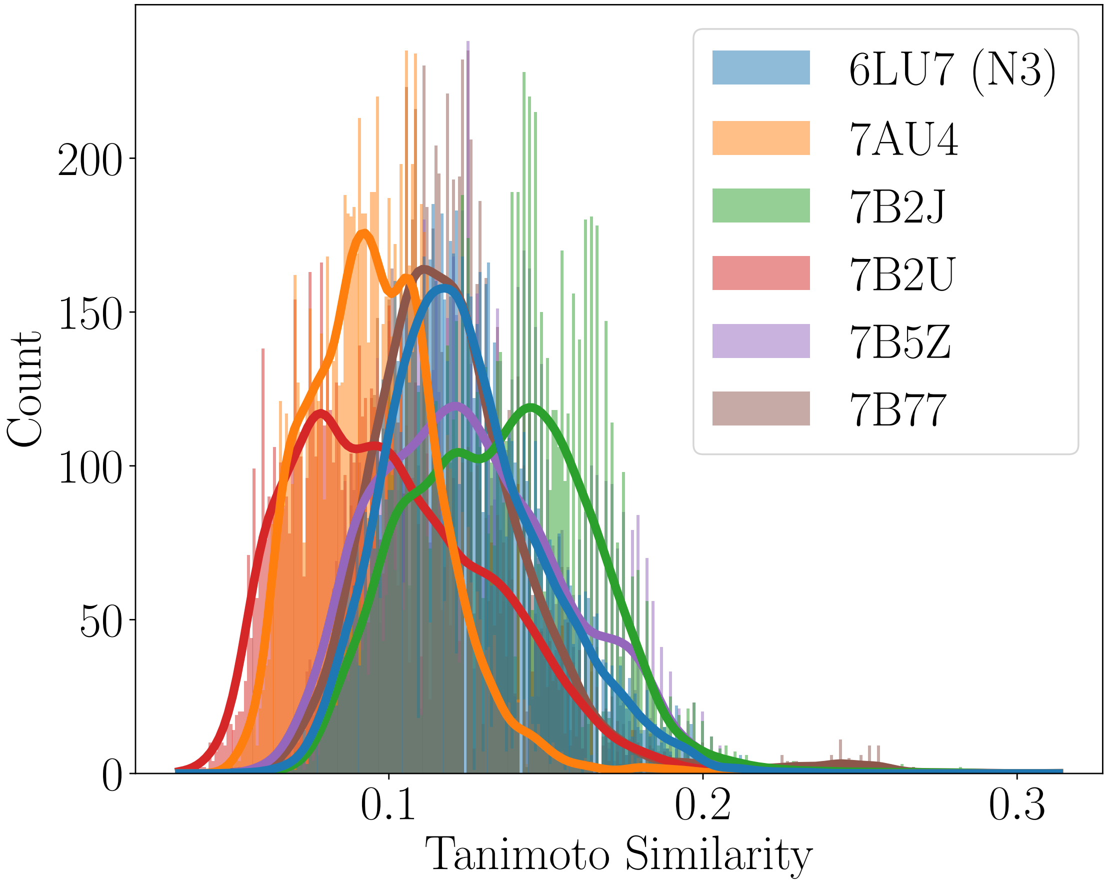

Figure S10: Pairwise similarity distribution among all designed molecules and the reference molecules from the bibliography. For simplicity and consistency, it is displayed the PDB accession code of each protein-ligand complex; however, only the ligand was used for the similarity calculations The 6LU7 (N3) inhibitor was obtained from Jin et al.[1], while the remaining references from Luttens et al.[6]. Tanimoto similarity was calculated using the Morgan fingerprint with a radius of 2 and 2048 bits. Solid lines depict smoothed histograms, computed using kernel density estimation (KDE) as described in the methods section. Tanimoto similarity ranges from 0 (no similarity) to 1 (identical molecules). These results emphasize that the 9,748 newly designed molecules exhibit low similarity to those reported in the aforementioned references

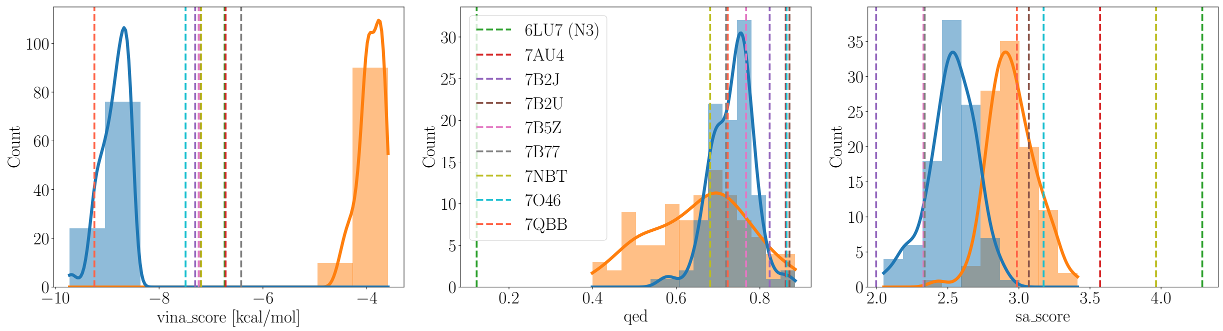

Figure S11: Distributions of AutoDock-Vina scores (*vina_score*, left panel), Quantitative Estimation of Drug-likeness scores (*qed*, middle panel) and Synthetic Accessibility scores (*sa_score*, right panel) for molecules of the final generation of the free (blue) and constrained (orange) campaigns. Solid lines depict smoothed histograms, computed using kernel density estimation (KDE). Dotted lines indicate property values of reference ligands calculated in this work. PDB accession codes are used as labels. 6LU7 (N3) structure was taken from Jin et al.[1], while the remaining from Luttens et al.[6]. Ligands 7NBT, 7O46, and 7QBB are the result of further optimization of hit structures identified during virtual screening, as reported by Luttens et al.[6]. Local optimization was allowed during docking for the reference ligands. The histograms were constructed using the Seaborn[5] Python library, with the number of bins automatically selected.

7QBB achieved the lowest *vina_score* from the Luttens et al.[6] inhibitors tested in this work, with a value of $-9.246\ \mathrm{kcal\,mol^{-1}}$. Notably, eight molecules from the final population of the free campaign exhibited lower *vina_score* values than 7QBB, with two molecules simultaneously outperforming 7QBB in terms of *vina_score*, *qed* and *sa_score*. These are encouraging results as the eight compounds (especially the optimized inhibitors 7NBT, 7O46, and 7QBB) proposed by Luttens et al.[6] were experimentally validated with good outcomes, suggesting that our designed molecules may also prove to be effective inhibitors.

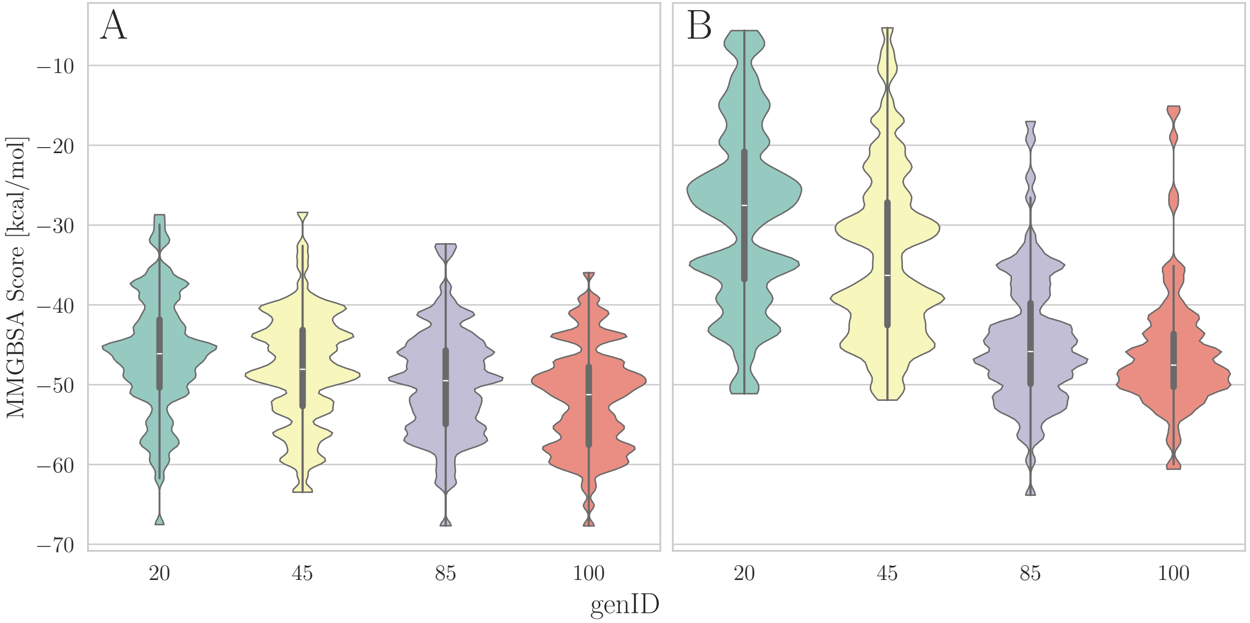

Figure S12: MMGBSA score distribution of the final population at each stage for the free (**A**) and constrained (**B**) campaigns. While each generation is designed to contain 100 molecules, exclusions were necessary in some cases. In generation 20 of the constrained campaign, only 69 unique molecules were present in the population. Additionally, in the free campaign, 4 molecules were excluded from MMGBSA calculations due to impracticality: 2 from generation 20, and 1 each from generations 45 and 100. Similarly, in the constrained campaign, 45 molecules were excluded: 23 from generation 20, 15 from generation 45, 4 from generation 85, and 3 from generation 100.

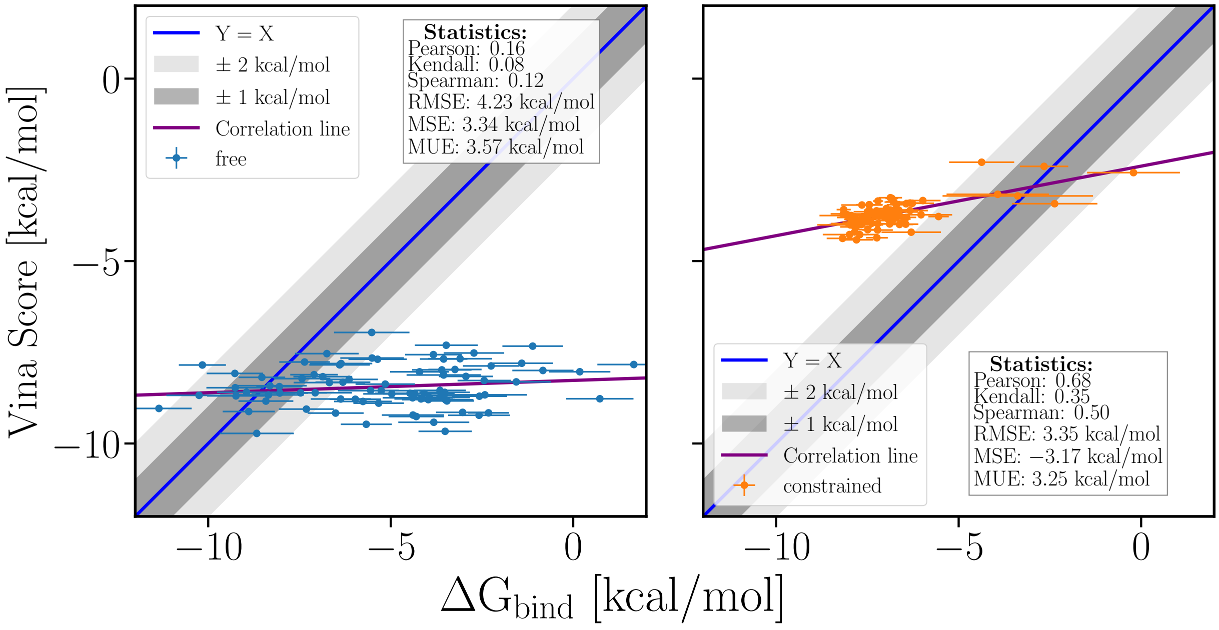

Figure S13: Correlation analysis between Vina scores and ABFEs for the 82 and 86 molecules submitted to free energy perturbation calculations from the free and constrained campaigns, respectively. Pearson, Kendall, and Spearman correlation coefficients are reported, along with root mean square error (RMSE), mean signed error (MSE), and mean unsigned error (MUE).

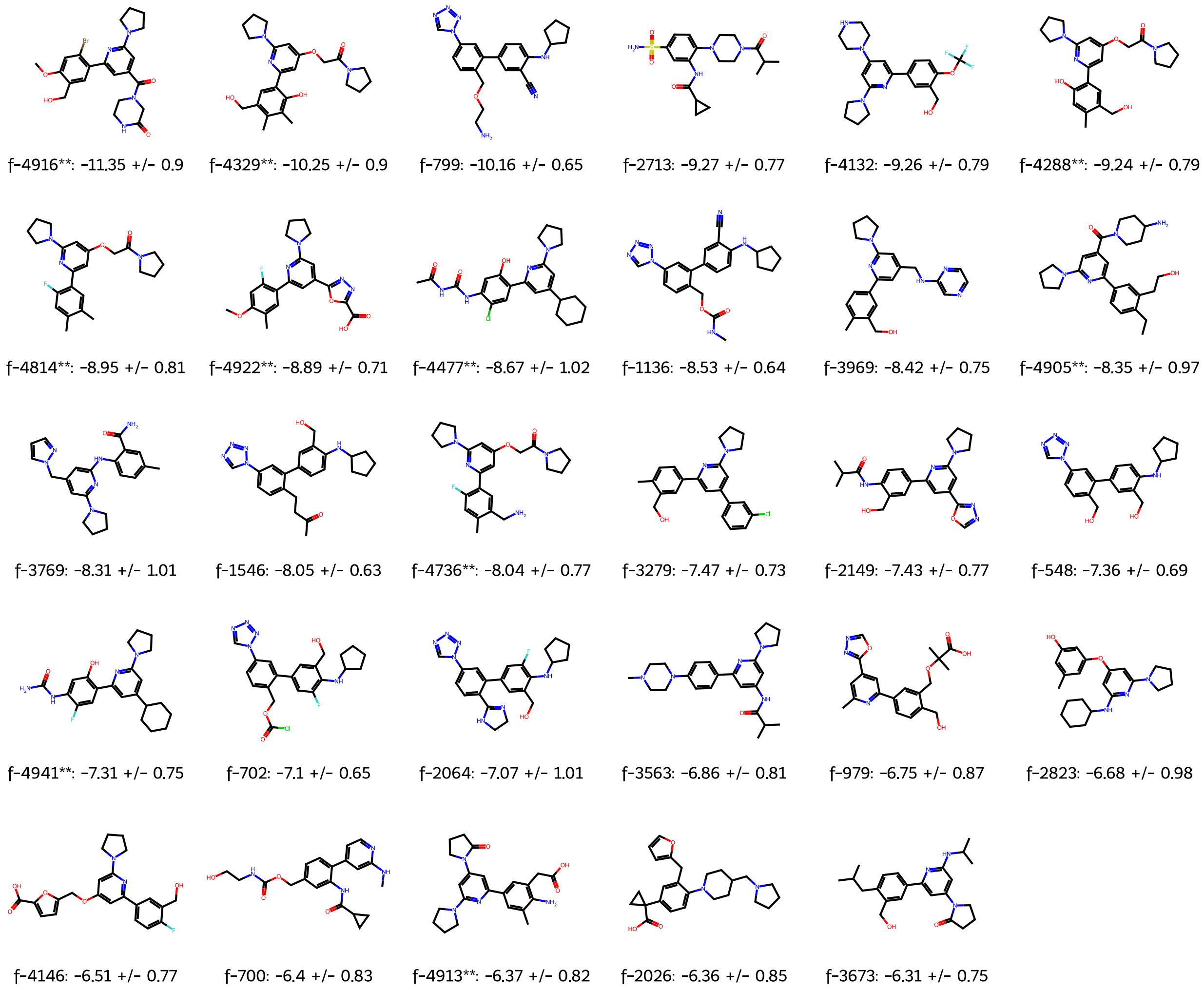

Figure S14: Molecules from the free campaign predicted to have better potency than the crystal structure molecule (N3). Molecules marked with “**” are highlighted as those present in the final generation. The estimated binding energy and its error are also reported, both in units of kcal mol^{-1}.

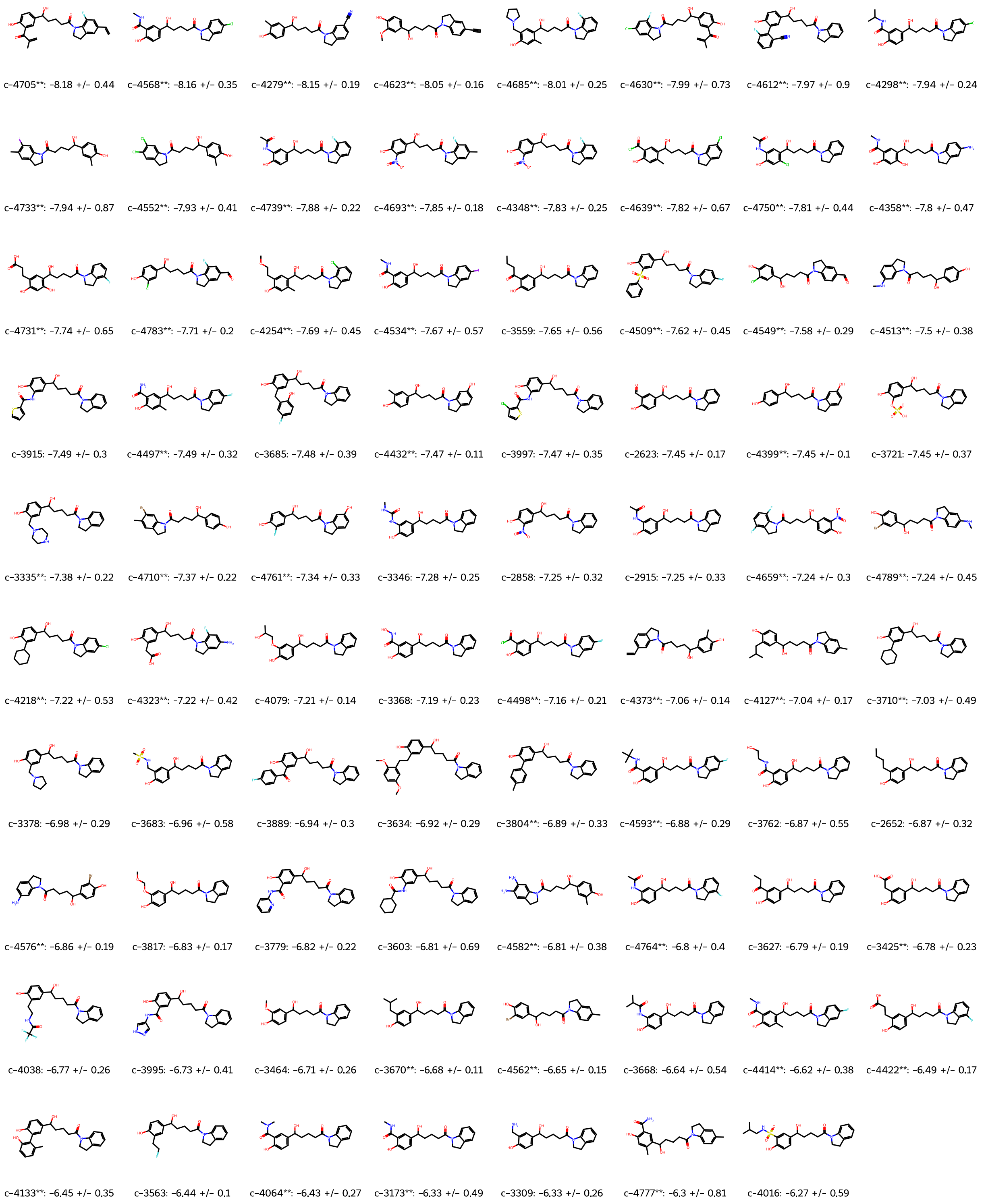

Figure S15: Molecules from the constrained campaign predicted to have better potency than the crystal structure molecule (N3). Molecules marked with "**" are highlighted as those present in the final generation. The estimated binding energy and its error are also reported, both in units of kcal mol^{-1}.

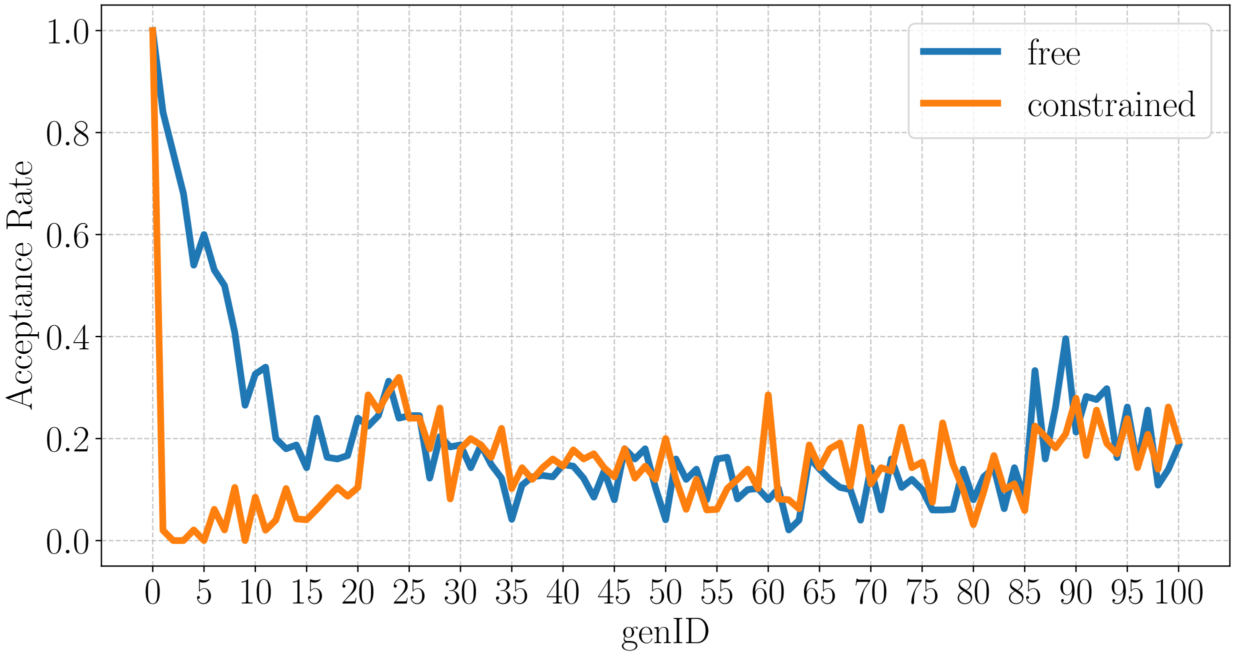

Figure S16: Acceptance rate over generations for the free and constrained campaigns.

Acknowledgement

We would like to thank Dr. Nils Weskamp and Dr. Miha Skalic for their insightful discussion and valuable inputs on the topic.

This research was funded by the European Union's Horizon 2020 Research and Innovation Program under Marie Skłodowska Curie Grant 860592.

Supporting Information Available

All configuration input files, output from the simulations and postprocessing scripts are available at https://doi.org/10.5281/zenodo.14237290.

Availability and requirements

- **Project name**: moldrug
- **Project home page**: https://moldrug.rtfd.io
- **Operating system**: Platform independent
- **Programming language**: Python
- **Other requirements**: rdkit>=2022.3.5, crem, tqdm, numpy, pandas, pyyaml, dill, meeko, six, scipy. All listed in the main project and automatically installed.
- **License**: Apache 2.0 license

References

(1) Jin, Z. et al. Structure of Mpro from SARS-CoV-2 and discovery of its inhibitors. *Nature* **2020**, *582*, 289–293.

(2) Landrum, G. et al. rdkit/rdkit: 2023-03-3 (Q1 2023) Release. 2023; `https://doi.org/10.5281/zenodo.8254217`.

(3) DeLano, W. L. The PyMOL molecular graphics system. *http://www. pymol. org/* **2002**,

(4) Bouysset, C.; Fiorucci, S. ProLIF: a library to encode molecular interactions as fingerprints. *Journal of Cheminformatics* **2021**, *13*, 1–9.

(5) Waskom, M. L. Seaborn: statistical data visualization. *Journal of Open Source Software* **2021**, *6*, 3021.

(6) Luttens, A. et al. Ultralarge Virtual Screening Identifies SARS-CoV-2 Main Protease Inhibitors with Broad-Spectrum Activity against Coronaviruses. *Journal of the American Chemical Society* **2022**, *144*, 2905–2920.
